# Physical inactivity and protein energy wasting play independent roles in muscle weakness in maintenance haemodialysis patients

**DOI:** 10.1371/journal.pone.0200061

**Published:** 2018-08-01

**Authors:** Jean-Sébastien Souweine, Nils Kuster, Leila Chenine, Annie Rodriguez, Laure Patrier, Marion Morena, Eric Badia, Lotfi Chalabi, Nathalie Raynal, Isabelle Ohresser, Helene Leray-Moragues, Jacques Mercier, Maurice Hayot, Moglie Le Quintrec, Fares Gouzi, Jean-Paul Cristol

**Affiliations:** 1 PhyMedExp, INSERM, CNRS, Univ Montpellier, Département de Biochimie et Hormonologie, CHU Montpellier, Montpellier, France; 2 Département de Néphrologie, CHU Montpellier, Univ Montpellier, Montpellier, France; 3 Département de Biochimie et Hormonologie, CHU Montpellier, Univ Montpellier, Montpellier, France; 4 AIDER, Montpellier, France; 5 PhyMedExp, INSERM, CNRS, Univ Montpellier, Département de Physiologie, CHU Montpellier, Montpellier, France; Universidade Estadual Paulista Julio de Mesquita Filho, BRAZIL

## Abstract

**Background:**

Muscle weakness is associated with increased mortality risk in chronic haemodialysis (CHD) patients. Protein energy wasting (PEW) and low physical activity could impair muscle quality and contribute to muscle weakness beyond muscle wasting in these patients. Aim of this study was to assess clinical and biological parameters involved in the reduction of muscle strength of CHD patients.

**Methods:**

One hundred and twenty-three CHD patients (80 males, 43 females; 68,8 [57.9–78.8] y.o.) were included in this study. Maximal voluntary force (MVF) of quadriceps was assessed using a belt-stabilized hand-held dynamometer. Muscle quality was evaluated by muscle specific torque, defined as the strength per unit of muscle mass. Muscle mass was estimated using lean tissue index (LTI), skeletal muscle mass (SMM) assessed by bioelectrical impedance analysis and creatinine index (CI). Voorrips questionnaire was used to estimate physical activity. Criteria for the diagnosis of PEW were serum albumin, body mass index < 23 kg/m^2^, creatinine index < 18.82 mg/kg/d and low dietary protein intake estimated by nPCR < 0.80g/kg/d.

**Results:**

MVF was 76.1 [58.2–111.7] N.m. and was associated with CI (β = 5.3 [2.2–8.4], p = 0.001), LTI (β = 2.8 [0.6–5.1], p = 0.013), Voorrips score (β = 17.4 [2.9–31.9], p = 0.02) and serum albumin (β = 1.9 [0.5–3.2], p = 0.006). Only serum albumin (β = 0.09 [0.03–0.15], p = 0.003), Voorrips score (β = 0.8 [0.2–1.5], p = 0.005) and CI (β = 0.2 [0.1–0.3], p<0.001) remained associated with muscle specific torque. Thirty patients have dynapenia defined as impaired MVF with maintained SMM and were younger with high hs-CRP (p = 0.001), PEW criteria (p<0.001) and low Voorrips score (p = 0.001), and reduced dialysis vintage (p<0.046).

**Conclusions:**

Beyond atrophy, physical inactivity and PEW conspire to impair muscle strength and specific torque in CHD patients and could be related to muscle quality.

**Trial registration:**

ClinicalTrials.gov NCT02806089

## Introduction

Sarcopenia, defined as skeletal muscle weakness associated with reduced muscle mass [1,2], appears as an emerging risk factor in chronic haemodialysis (CHD) patients due to high prevalence [3,4] and increased mortality [5]. Thus, identifying factors of sarcopenia is highly warranted in CHD patients. Most studies have focused on determinants of muscle mass in CHD patients. By contrast, muscle strength determinants have been poorly investigated in this population whereas muscle strength appears as a better mortality prognosis factor than muscle mass [3]. In addition, it influences the independence in activities of daily living in these patients [6]. It has been generally assumed that muscle mass was the main determinant of muscle strength [6]. However, malnutrition and other factors including inflammation, metabolic acidosis, insulin resistance, hormones and uremic milieu may also be involved in muscle weakness of CHD patients [7–9]. All these factors, which are related to protein energy wasting (PEW), characterized by a loss of systematic proteins, an hypercatabolic status, uremic toxins, malnutrition [10,11], could play a role in both muscle mass and strength reduction. In addition, muscle quality, evaluated by muscle specific torque (defined as strength per unit of muscle mass [12]), may also impact muscle strength [13]. Loss of muscle strength which is not related to decrease in muscle mass has been defined as dynapenia [14,15]. Impairment in muscle capillarisation, fibre type distribution, mitochondrial energy and glycogen reserves [16,17] have been reported in CHD patients. Factors like physical activity reduction, leading to deconditioning, can also be involved in muscle structural impairment. As a working hypothesis, we postulated that beyond atrophy, factors of muscle quality like physical inactivity and PEW conspire to weaken muscle strength and specific torque in CHD patients. Yet, the relative contribution of these factors on the onset and maintenance of muscle weakness in CHD patients should be assessed [8,18] and appears as a pre-requisite for designing specific therapeutic interventions [11].

Therefore, the aim of this study was to assess the clinical and biological parameters involved in the reduction of the muscle strength in CHD patients. In addition, in order to highlight the potential determinants of muscle strength beyond muscle wasting, we aimed to identify determinants of specific torque.

## Materials and methods

### Ethics statement

The study was conducted according to the principles of the Declaration of Helsinki and in compliance with International Conference on Harmonization/Good Clinical Practice regulations. The research protocol was approved by the institutional ethics committee of Marseille University Hospital (Comité de Protection des Personnes Sud Méditerranée I) in January 2016 with the following number 2015-A01854-45. The authors confirm that all ongoing and related trials for this drug/intervention are registered (ClinicalTrials.gov Identifier: NCT02806089). However, registration of the study on ClinicalTrials.gov was done after recruitment began. This registration was not a pre-condition necessary to start a trial in France. All patients gave their written informed consent.

### Patients

**Fig 1:**
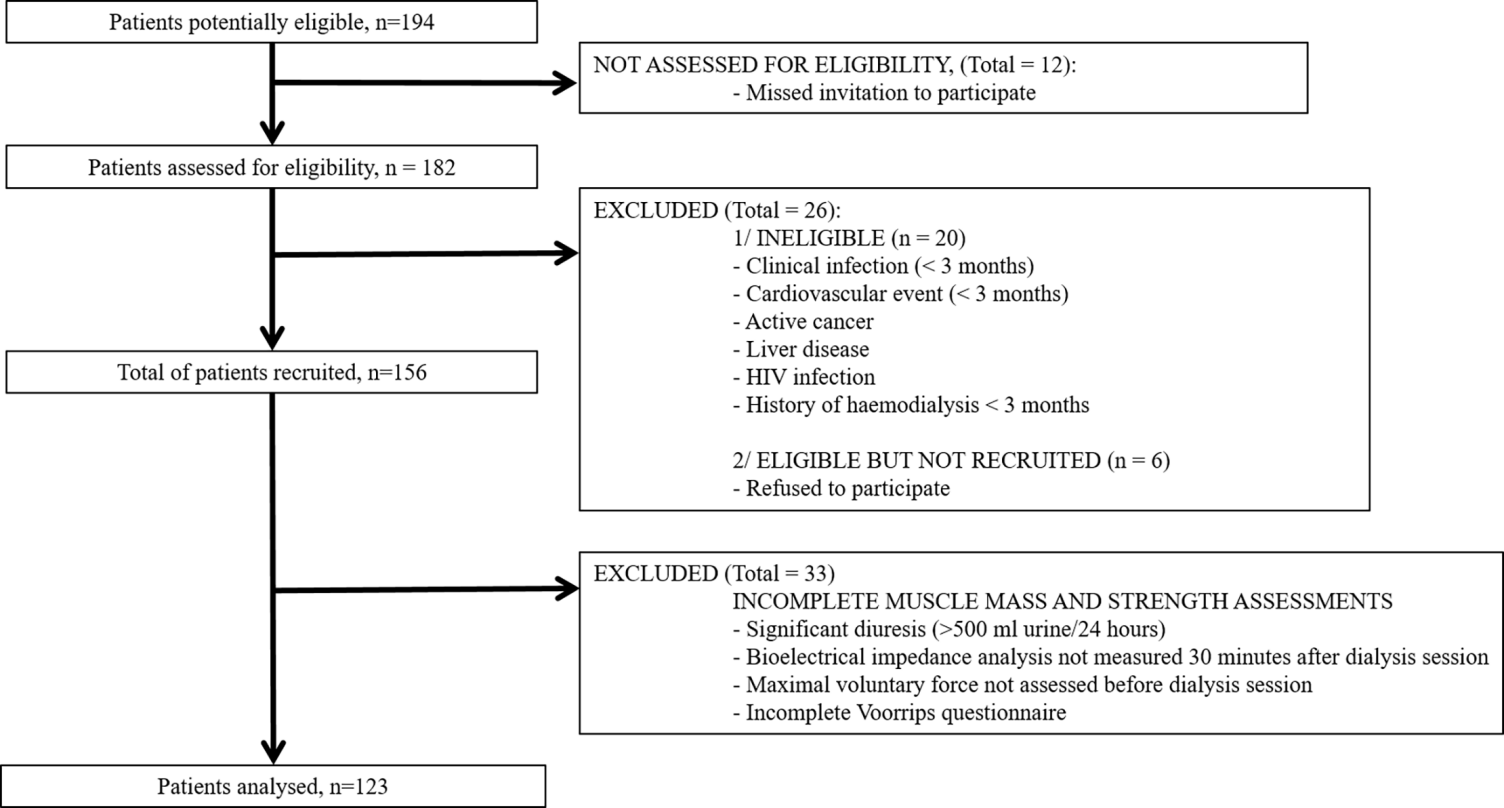
Flow chart diagram depicting number of patients evaluated for eligibility and number of patients included in analysis.

Chronic haemodialysis patients were enrolled in 4 haemodialysis units of Languedoc Roussillon, France (Lapeyronie University Hospital and 3 centers issued from a non-profit dialysis association [AIDER]). Screening period started in September 2015 until end of December 2015. During this period, investigators were trained to muscle mass, strength, and physical activity measurement tools. Then, after ethics committee approval, participants were enrolled from January 2016 to June 2016 (Fig 1).

Prevalent adult haemodialysis patients, with a dialysis schedule of 3 sessions per week more than three months could be included in this study. CHD patients were not included if they had clinical infection or cardiovascular event during the last three months before the inclusion, active cancer, liver disease, or HIV infection at the time of evaluation. Patients who had measurement bias in muscle mass or strength evaluations were excluded from the analysis [13,19,20].

### Procedures

Clinical examination, biological parameters, muscle mass and strength measurements were performed the day of inclusion. Maximal voluntary force of quadriceps was assessed before dialysis session, and bioelectrical impedance analysis was assessed after the same dialysis session. Medical records were reviewed for age, gender, pre- and post-dialysis weight, treatment modalities, duration of kidney disease and dialysis vintage. History of comorbidities was performed using Charlson score for each patient [21]. Pre- and post-dialysis blood samples were collected during a mid-week dialysis session. Serum high-sensitivity C-reactive protein (hs-CRP) and serum albumin were determined by immunoturbidimetry (Cobas 8000, Roche, Meylan, France). Dialysis adequacy was estimated by calculation of Daugirdas single pool equation (_sp_Kt/V urea) [22]. Body mass index (BMI) was obtained using post-dialysis weight. Normalised protein catabolism rate (nPCR) was calculated from pre- and post-dialysis blood urea and dialysis adequacy (_sp_Kt/V urea) [23].

### Muscle mass determination

Body composition was firstly estimated by Bioelectrical impedance analysis (BIA) using the body composition monitor (BCM, Fresenius Medical Care, Bad Homburg,Germany) [20] thirty minutes after dialysis session. BCM is a three compartment model, giving lean tissue mass (LTM), fat tissue mass (FTA) and overhydration. Moreover, resistance is measured at a frequency of 50kHz in order to calculate skeletal muscle mass (SMM) [5,24]. The formula for SMM was as follows:

$$SMM=\left[(\frac{Height^{2}}{Resistance})\;x\;0.401\right]+(Gender\;x\;3.825)+(Age\;x\;(-0.071))+5.102$$

SMM = skeletal muscle mass in kg; height in centimetres; resistance in ohms; gender: women = 0, men = 1; age in years.

Then, lean tissue mass, fat tissue mass and muscle mass (kg), were normalized for squared height and defined as lean tissue index (LTI), fat tissue index (FTI) and skeletal muscle mass index (SMMI).

Creatinine index (CI), a well-known marker of muscle mass in CHD patients[19], was also assessed. CI, defined as the normalized creatinine production rate, equals the sum of creatinine excretion rate (dialytic removal and urinary excretion) and metabolic degradation rate in the steady state [19]. CI equation excludes patients with a significant diuresis (>500 ml urine/24 hours) and/or residual renal function (>2 ml/min). Therefore, patients with a significant diuresis (>500 ml urine/24 hours) and/or residual renal function (Glomerular Filtration Rate >2 ml/min) were excluded from the analysis [19]. The cutoff point of 18.82 mg/kg/d has been used as CI value <18.82 mg/kg/d is significantly associated with mortality [25].

### Protein energy wasting evaluation

Criteria for the clinical diagnosis of PEW were ≥3 out of the 4 items: serum albumin < 38g/l, body mass index < 23 kg/m^2^, creatinine index < 18.82mg/kg/d, low dietary protein intake estimated by nPCR < 0.80g/kg/d [10,25].

### Muscle strength and physical activity assessment

Assessment of maximal voluntary force of quadriceps using a dynamometer chair represents the current recommended method for screening muscle weakness [26,27]. Maximal voluntary force was a join torque (Newton.meter) which was calculated by multiplying quadriceps strength (in Newton) by the lever arm length, defined as the distance from the meniscuses to the leg fastening zone, in meter. Recently, we validated in healthy volunteers and CHD patients a new tool to assess MVF of quadriceps at the patients’ bedside before dialysis session in the dominant leg using a belt-stabilized hand held dynamometer (HHD) (Microfet 2 (Hogan Health Industries, Inc West Jordan) [13].Subsequently, the normative database of MVF from French adults obtained with the dynamometer chair can be applicable to our CHD patients [28] and results obtained in dialysis population were compared to the theoretical quadriceps strength calculated using the predictive regression model [28]. In order to explore muscle quality, a specific torque has been defined as the ratio of MVF of dominant quadriceps by SMM [29]. Physical activity was assessed using Voorrips questionnaire which was validated in CHD patients [30,31]. A Voorrips score below 9.4 defined low physical activity [30].

### Statistical analyses

Sample size calculation: The study was designed to detect a decrease in maximal voluntary force of quadriceps for haemodialysis patients who are exposed to physical inactivity and protein energy wasting. Sample size was computed assuming a multivariable model with 5 covariates (albumin, hs-CRP, Voorrips score, Creatinine Index and LBM). Assuming a type I error alpha = 5%, a type II error (1-power) of beta = 10% and a medium effect size of 0.15 as proposed by Cohen [32], the needed sample size to identify main parameters involved in muscle weakness in this cross-sectional study was 115 patients.

Population characteristics were expressed as median (quartile 1-quartile 3) for quantitative variables and as proportions for categorical variables. Logarithm transformations were performed for Voorrips score and hs-CRP data to obtain a normal sampling distribution. Comparisons were performed using Mann-Whitney U-test, and Kruskal-Wallis test for quantitative data and Chi-squared test for categorical data. Association between MVF and patient characteristics were quantified using linear regression. Results were expressed as beta coefficients [95% confidence interval]. Variables significant at α = 0.2 level in univariate analysis were subsequently tested in multivariate analysis. A stepwise procedure using Akaike Information criterion (AIC) was used to select potential variables in the final model. This criterion is based on the likelihood of the model penalized for model complexity. Validity of the linear regression model was tested via visual inspection of residuals. Shapiro-Wilk test was used to test normality of residuals. Breusch-Pagan test was used to check the homoscedasticity of the model. To avoid bias related to location of enrollment, models were adjusted for enrollment centre. Potential collinearity problems in the final models were assessed via computation of Variance Inflation Factors (VIF). In order to speculate on the potential relationships between the different significant determinants in multivariate analysis, partial correlations that measure the degree of association between two random variables after excluding the effect of all other covariates were performed. Partial Spearman’s correlations were computed using the ppcor package for R software. In order to further determine the determinants of muscle quality, the multivariate analysis was also performed using the specific torque as end-point. Quantile regression was used to further explore the relationship between muscle mass and muscle strength. Dynapenia was defined as muscle strength below the 25^th^ percentile for a given muscle mass. All statistical analyses were performed using R 3.1.0 software (The R Project for statistical computing, www.r-project.org). *P-*values of <0.05 were considered to be statistically significant.

## Results

### Characteristics of the studied population

One hundred and twenty-three CHD patients (80 males, 43 females; 68.8 [57.9–78.8] y.o.) were included in the study (Fig 1). Causes of end-stage renal disease were glomerulonephritis (23.4%), diabetes mellitus (20.4%), hypertensive nephrosclerosis (14.6%), others (25.4%) and undetermined nephropathies (16.2%).

**Fig 2:**
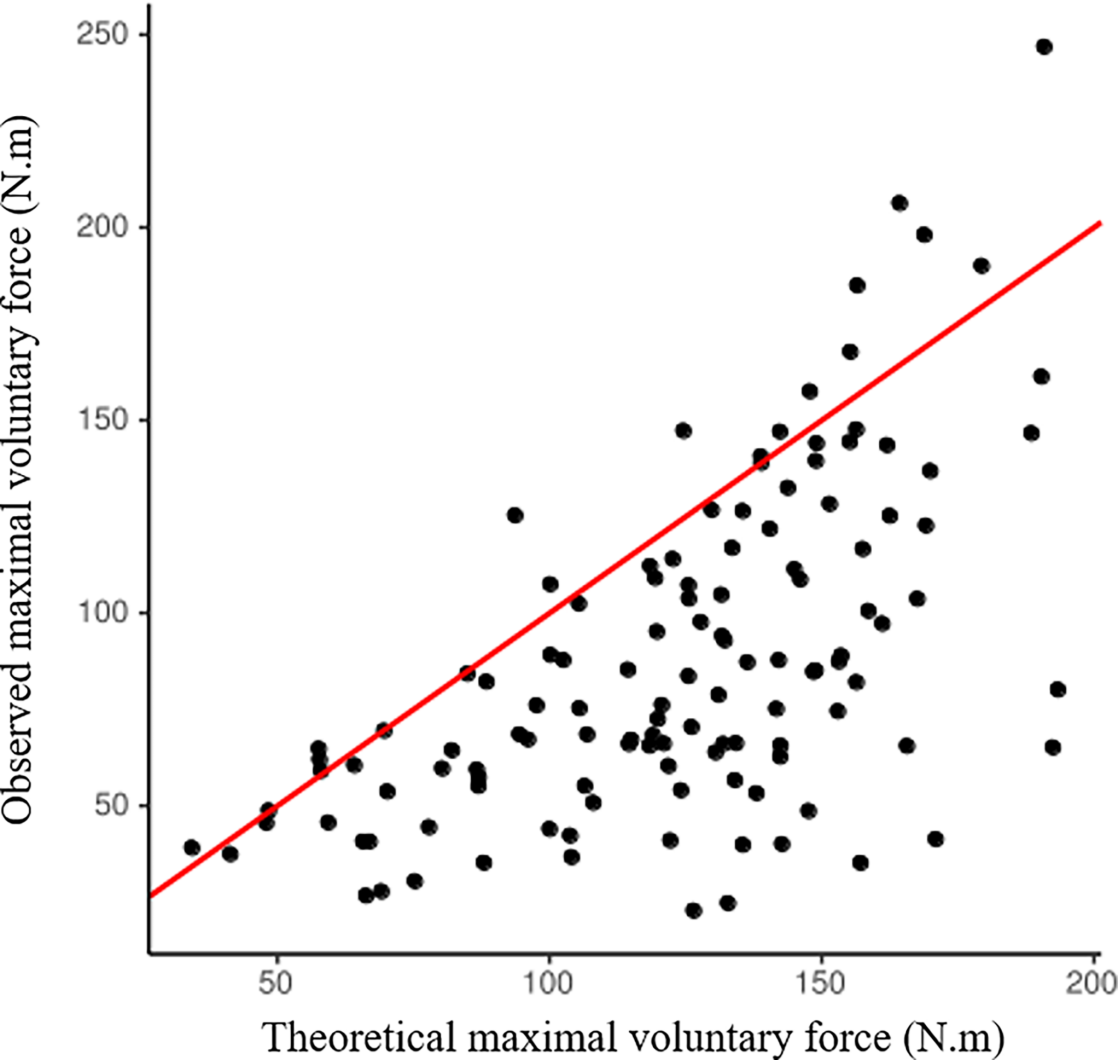
Correlation between observed maximal voluntary force (N.m) and theoretical maximal voluntary force (N.m). Red line represents the line of identity.

Clinical and biological characteristics of the patients are summarized in Table 1. Charlson score was 6 [4–7], _sp_Kt/V urea was 1.8 [1.5–1.9] and dialysis vintage was 2,9 [1.0–7.2] years). Maximal voluntary force ranged from 22.7 to 246.8 N.m with a median at 76.1 [58.2–111.7] N.m. As shown in Fig 2, according to the French isometric strength normative database, CHD patients presented lower MVF levels than expected values. The mean difference between observed and theoretical values was—28.3% ± 25.8%. In addition, a reduction in physical activity was also observed in these patients, as demonstrated by low Voorrips scores (5.0 [2.9–7.7]) (Table 1).

**Table 1:** Patient characteristics according to dialysis centers. Values were described by using proportions for categorical variables and median (range) for quantitative variables. Centers: #1 and #2 were dialysis centers while #3 and #4 were self care dialysis units.*(BMI*: *body mass index; BP*: *blood pressure; CKD chronic kidney disease; Hb*: *haemoglobin; hs-CRP*: *serum high-sensitive C-reactive protein; LTI*: *lean tissue index; MVF*: *maximal voluntary force; nPCR*: *normalised protein catabolism rate; SMM*: *skeletal muscle mass; SMMI skeletal muscle mass index*.*)*.

Parameters	Total Population	Center 1	Center 2	Center 3	Center 4	p
	n = 123	n = 41	n = 23	n = 34	n = 25	
*Demographic and clinical characteristics*						
Age (y)	68.8 (57.9–78.8)	73.8 (67.2–81.9)	61.7 (44.5–67.8)	68.4 (59.7–79.9)	68.1 (35.0–76.4)	**0.001**
Gender, Men, n (%)	80 (65%)	26 (63.4%)	19 (82.6%)	18 (52.9%)	17 (68%)	0.141
sp KT/V	1.8 (1.5–1.9)	1.8 (1.6–2.0)	1.61 (1.3–1.8)	1.75 (1.5–1.9)	1.8 (1.6–2.0)	**0.024**
Charlson score	6.0 (4.0–7.0)	7 (6–8)	5 (3.5–6)	5.5 (4–7.7)	5 (4–7)	**< 0.001**
Duration of CKD (y)	10.2 (4.9–19.5)	6.3 (3.5–13.2)	12.3 (4.2–18.0)	7.7 (4.7–17.2)	19.5 (13.4–22.6)	**0.001**
Dialysis vintage (y)	2.9 (1.0–7.2)	1.4 (0.3–2.7)	1.6 (0.6–3.3)	4.5 (2.5–10.1)	7.1 (4.7–11.5)	**< 0.001**
BMI (kg/m^2^)	24.2 (21.3–27.1)	24.5 (21.0–27.4)	24.5 (23.0–27.0)	25.8 (23–29.3)	23.7 (21.4–26.3)	0.364
Predialysis systolic BP (mmHg)	128 (113–148)	116 (107–135)	135 (125–152.5)	127.5 (109.2–151.7)	133 (119–147)	0.027
Predialysis diastolic BP (mmHg)	64 (53–77)	55 (48–66)	66 (55–76.5)	61.5 (50.25–77.5)	76 (64–80)	0.003
FTI (kg/m^2^)	12.1 (8.6–16.3)	10.8 (7.8–16.3)	13.1 (6.6–15.4)	15.6 (12.2–19.3)	10.4 (9.5–13.2)	**0.007**
LTI (kg/m^2^)	11.5 (9.7–13.4)	11.7 (9.7–13.7)	11.6 (10.1–14.7)	10.0 (8.3–11.1)	12.1 (11.6–13.3)	**0.004**
SMM (kg)	22.0 (17.0–25.5)	23.6 (17.1–26.5)	23.9 (22.0–26.6)	19.9 (14.5–21.9)	22.9 (18.5–27.1)	**0.004**
SMMI (kg/m^2^)	7.8 (6.6–8.6)	8.0 (6.7–8.9)	8.1 (7.8–8.8)	6.8 (6.0–7.5)	8.2 (6.9–8.8)	**0.001**
Voorrips score	5.0 (2.9–7.7)	0.5 (0.2–0.7)	0.73 (0.5–0.8)	0.7 (0.4–1.0)	0.8 (0.7–0.9)	**0.005**
MVF (N.m)	76.1 (58.2–111.7)	64.7 (45.6–88.8)	84.7 (59.7–103.6)	74.5 (56.8–122.4)	97.65 (75.1–140.6)	0.003
*Protein Energy Wasting*, presence, n (%)	53 (43%)	22 (53.7%)	10 (43.5%)	9 (26.5%)	2 (8%)	**0.001**
*Laboratory parameters*						
Serum albumin (g/l)	38.3 (35.4–41.0)	35.4 (32.9–37.5)	37 (34–41)	39.85 (38.2–41.6)	41 (39–42)	**< 0.001**
hs-CRP (mg/l)	4.0 (2.1–7.9)	0.5 (0.3–0.8)	0.7 (0.5–1.1)	0.6 (0.4–0.8)	0.6 (0–0.85)	0.161
nPCR (g/kg/j)	0.8 (0.7–1.02)	0.8 (0.6–0.9)	0.8 (0.7–1.0)	0.9 (0.7–1.0)	0.8 (0.7–1.0)	0.143
Serum bicarbonate (mmol/l)	22.4 (21.0–25.0)	25 (23–26)	22 (20.5–23.0)	22.3 (20.8–23.65)	22 (21–25)	0.001
Hb (g/dl)	11.3 (10.6–12.0)	11.3 (9.5–12.0)	11.3 (10.6–11.9)	11.3 (10.8–11.9)	11.4 (10.5–12.0)	0.848
Creatinine index (mg/kg/j)	18.7 (17.2–20.6)	17.5 (16.1–18.7)	19.8 (18.5–21.3)	18.9 (17.3–20.5)	20.3 (18.7–22.4)	**< 0.001**

### Determinants of muscle strength

In univariate analysis, (Table 2), age, dialysis center and Charlson score were significantly associated with decreased muscle strength whereas no association was reported with duration of chronic kidney disease (CKD) and dialysis vintage. MVF was also associated with muscle mass estimated from CI (β = 9.8 [7.8–11.9], p <0.001), SMI (β = 14.2 [9.7–18.6] p<0.001) and LTI (β = 8.0 [5.6–10.3] p<0.001) and with Voorrips score (β = 58.4 [39.3–77.4], p<0.001). MVF was associated i) positively with serum albumin (β = 4.0 [2.4–5.6] p<0.001), ii) negatively with hs-CRP (β = -17.7 95% CI [-34.5– - 0.9] p = 0.04). Finally, no association with daily protein intake assessed by nPCR was observed (p = 0.467).

**Table 2:** Univariate analysis of variables associated with maximal voluntary force. (hs-CRP: serum high-sensitive C-reactive protein; LTI: lean tissue index; nPCR: normalised protein catabolism rate; SMM: skeletal muscle mass; SMMI skeletal muscle mass index).

Variable		Coefficient (95% CI)	p
Age		-1.0 (-1.4–0.6)	p < 0.001
Gender: Men vs Women		38.3 (24.0–52.6)	p < 0.001
Voorrips score		58.4 (39.3–77.4)	p < 0.001
Charlson score		-7.3 (-10.3–4.3)	p < 0.001
Duration of CKD		0.1 (-0.2–0.5)	p = 0.534
Dialysis vintage		-2.2 (-14.0–9.5)	p = 0.711
sp KT/V		-43.1 (-62.5–23.7)	p < 0.001
Body mass index		0 (-1.5–1.5)	p = 0.996
nPCR		11.9 (-20.1–43.9)	p = 0.467
Creatinine index		9.8 (7.8–11.9)	p < 0.001
Serum albumin		4.0 (2.4–5.6)	p < 0.001
PEW: Absence vs Presence		-20.7 (-36.1–5.2)	p = 0.01
hs-CRP		-17.7 (-34.5–0.9)	p = 0.04
Serum bicarbonate		-2.0 (-5.0–1.0)	p = 0.203
Urea		1.6 (0.3–2.9)	p = 0.018
Haemoglobin		-1.5 (-7.2–4.1)	p = 0.604
Lean tissue index		8.0 (5.6–10.3)	p < 0.001
Skeletal muscle mass		14.2 (9.7–18.6)	p < 0.001
Skeletal muscle mass index		4.1 (3.0–5.1)	p < 0.001
Location of enrollment			
	Center 1	Reference	Reference
	Center 2	15.2 (-5.7–36.1)	p = 0.157
	Center 3	20.2 (1.6–38.9)	p = 0.035
	Center 4	36.3 (15.9–56.7)	p = 0.001

In AIC-based multivariate modelling, (Table 3); after adjustment for age, gender, dialysis location and dialysis vintage, muscle mass estimated from CI, LTI or SMM was highly associated with MVF (β = 5.3 [2.2–8.4], p<0.001; β = 2.8 [0.6–5.1], p = 0.013; β = 2.5 [0.9–4.1], p = 0.03 respectively). Voorrips score and serum albumin remained positively associated with MVF (β = 17.4 [2.9–31.9], p = 0.02 per one log increase and β = 1.9 [0.5–3.2], p = 0.006, respectively). By contrast, only CI (p<0.001), serum albumin (p<0.003) and Voorrips score (p = 0.005) were significantly associated with specific torque after adjustment for age, gender, dialysis location, and dialysis vintage.

**Table 3:** Multivariate analyses of (A) main determinants of maximal voluntary force and of (B) variables associated with muscle torque. (after adjustment for age, gender, dialysis center and dialysis vintage).

Variable	Coefficient (95% CI)	p
**Main determinants of maximal voluntary force**		
Lean tissue index	2.8 (0.6–5.1)	0.013
Serum albumin	1.9 (0.5–3.2)	0.006
Voorrips score	17.4 (2.9–31.9)	0.02
Skeletal muscle mass	2.5 (0.9–4.1)	0.003
Creatinine index	5.3 (2.2–8.4)	0.001
**Variables associated with muscle torque**		
Serum albumin	0.09 (0.03–0.15)	0.003
Voorrips score	0.89 (0.28–1.5)	0.005
Creatinine index	0.25 (0.12–0.38)	0.001

**Fig 3:**
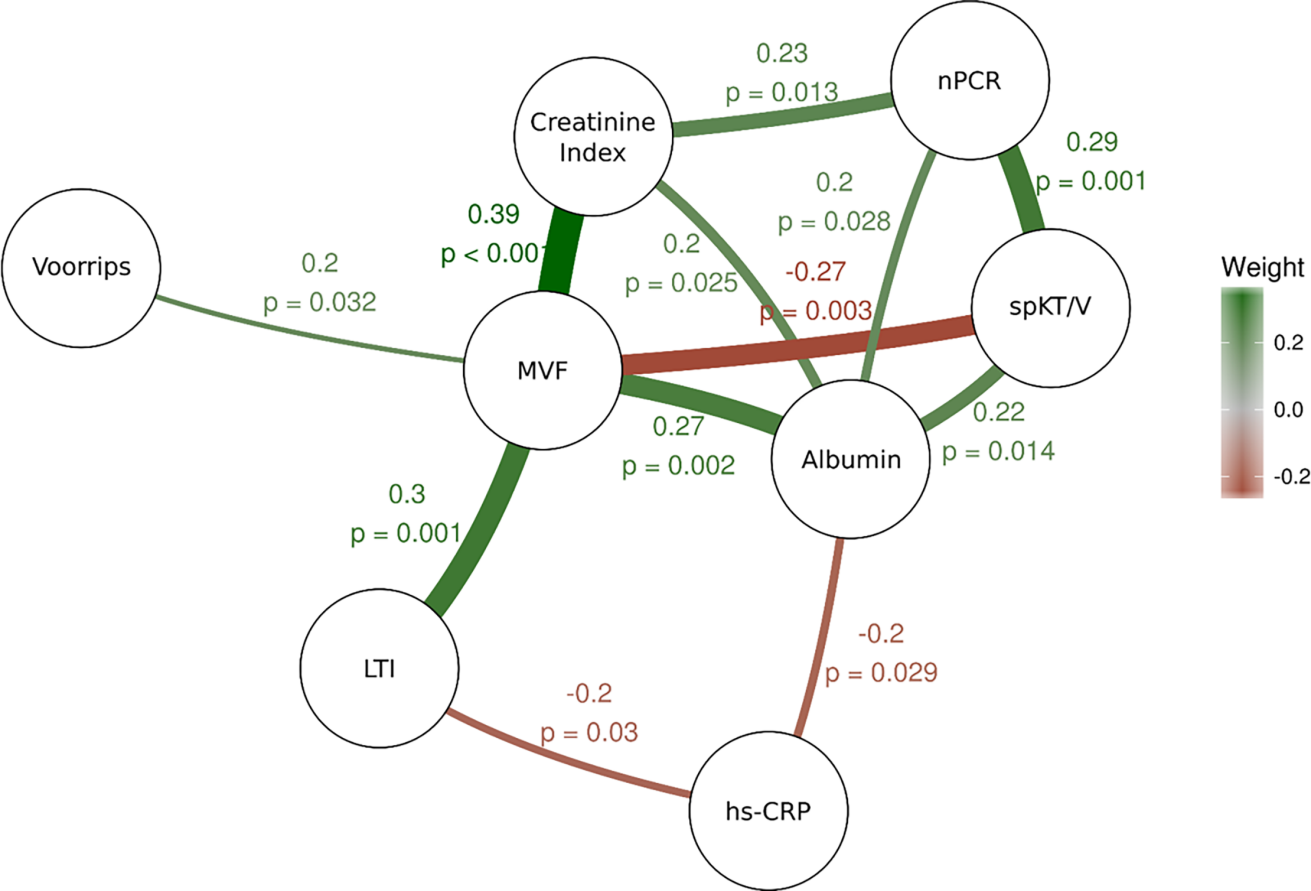
Spearman’s partial correlation. The correlation network shows partial correlations of maximal voluntary force, muscle mass, nutritional markers and dialysis dose.

Partial correlation between MVF and the different significant determinants was graphically represented on Fig 3. Muscle mass expressed as LTI or CI was closely linked to MVF. Inflammation assessed by hs-CRP was negatively associated with LTI (ρ = -0.21 p = 0.0024) and serum albumin (ρ = -0.22 p = 0.018) which in turn was linked to MVF. Dialysis adequacy appeared negatively associated with MVF, (ρ = -0.27 p = 0.003).

### Identification of dynapenia

**Fig 4:**
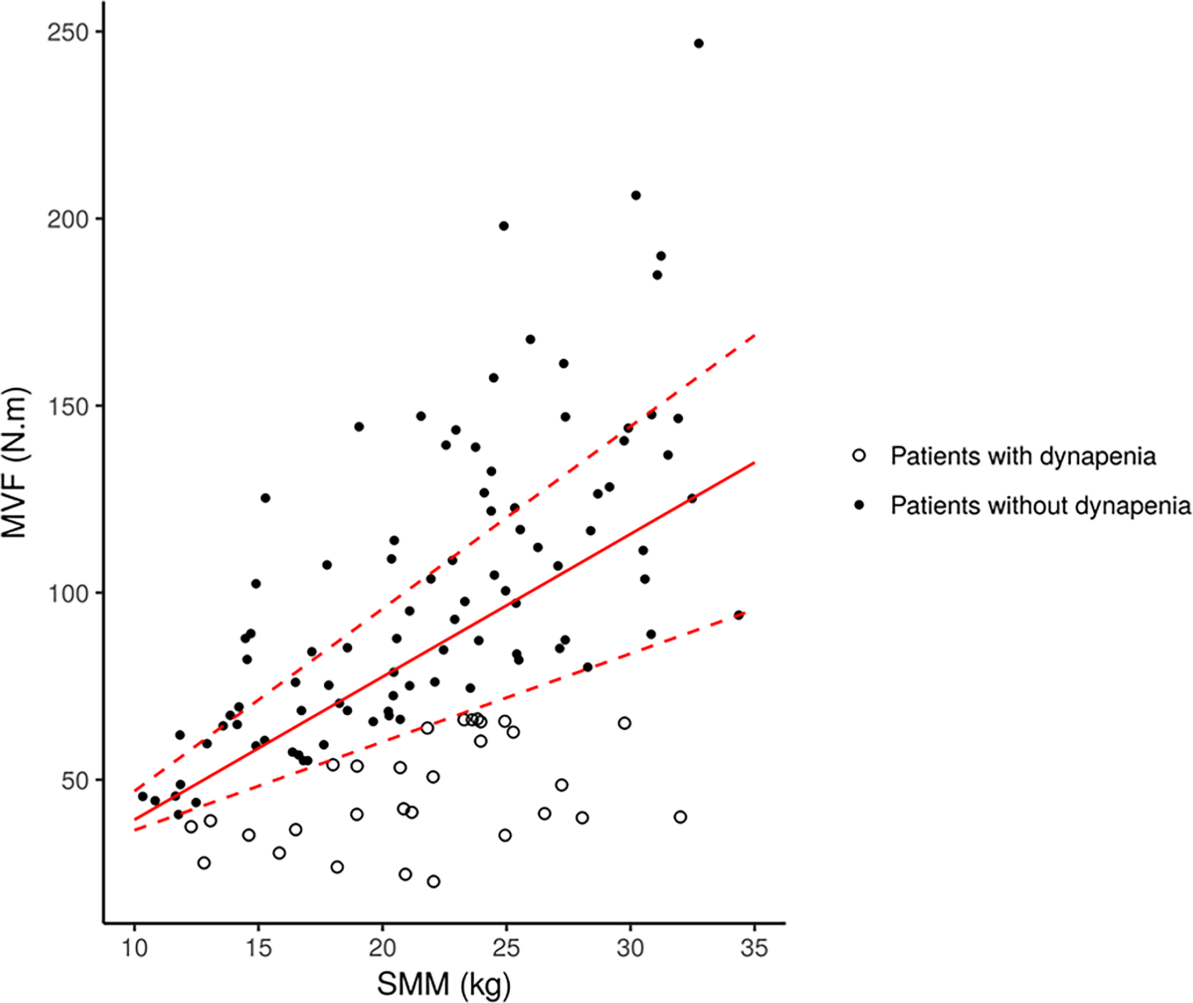
Quantile distribution between maximal voluntary force and skeletal muscle mass. Dynapenia was defined as the lowest quartile.

In order to explore the relationship between muscle strength and muscle mass, a quantile regression analysis was performed for first, second and third quartile (Fig 4). From this plot, we note an increase in muscle strength dispersion with muscle mass. Dynapenia, corresponding to patients with low MVF but maintained muscle mass, was defined as the lowest quartile of force for a given mass. In addition, characteristics of CHD patients with dynapenia were compared to all others patients. Patients with muscle weakness but preserved muscle mass were older with PEW, high comorbidities, high grade inflammation and low physical activity. (Table 4).

**Table 4:** Discordance between maximal voluntary force and skeletal muscle mass. (CKD chronic kidney disease; hs-CRP: serum high-sensitive C-reactive protein; MVF: maximal voluntary force; nPCR: normalised protein catabolism rate).

Variable	Patients with dynapenia (n = 30)	Other patients (n = 93)	p
Age (y)	75.1 (66.3–80.7)	67.7 (55.2–76.9)	**0.02**
Gender, Men, n (%)	19 (63.3%)	61 (65.6%)	0.9
Charlson score	7 (6–8)	6 (4–7)	**0.001**
sp KT/V	1.84 (1.5–2.0)	1.7 (1.5–1.9)	0.2
Duration of CKD (y)	10.4 (3.6–19.3)	10.2 (5.2–19.5)	0.4
Dialysis Vintage (y)	2.25 (1.21–7.14)	3.4 (1.0–7.1)	**0.4**
Voorrips score	0.5 (0.2–0.7)	0.7 (0.5–0.9)	**0.001**
Serum albumin (g/l)	35.4 (32.9–36.9)	39.2 (37.0–41.7)	**< 0.001**
hs-CRP (mg/l)	0.7 (0.5–1.0)	0.6 (0.2–0.8)	**0.01**
nPCR (g/kg/j)	0.8 (0.7–1.0)	0.8 (0.7–1.0)	**0.3**
Protein Energy Wasting n (%)	17 (56.7%)	26 (28%)	**0.008**
Serum bicarbonate (mmol/l)	23.5 (22.2–25.4)	22.4 (21–25)	0.06

## Discussion

In our population of 123 CHD patients, the main determinants of muscle strength were low muscle mass (determined either with BIA or CI), reduced physical activity and low serum albumin. Moreover, the muscle mass-independent analysis of muscle strength indicates that malnutrition, low physical activity and inflammation may specifically impact muscle quality in CHD patients.

We observed here that MVF was dramatically lower than theoretical MVF in our CHD patients. This result, of almost 30% reduction in MVF, appears in line with the magnitude of muscle weakness reported in large cohorts of CHD patients [3]. Creatinine index, LTI and SMM were associated with muscle strength. These findings confirm that muscle mass is a strong determinant of muscle strength. In addition CI but not LTI was significantly associated with muscle specific torque. Although a correlation was observed between CI and LTI (Spearman’s rho = 0.390, p<0.001), no multicollinearity problem could be evidenced in the final model (VIF<5, data not shown). This result supports the fact that BIA measures global muscle mass considering muscle tissue heterogenity such as fibrosis and adipose tissue [33] whereas CI is influenced by muscle protein turnover [34]. In line with this observation, we could hypothesize that CI is a dynamic indicator of muscle functionality. On the other hand, in the Spearman partial correlation, dialysis quantification using _sp_KT/V was negatively associated with decreased muscle strength (Fig 3). This result may be explained by the fact that _sp_KT/V, is inversely related to urea and water volume distribution [35] mainly related to muscle mass [36]. Thereby, the _sp_KT/V increase may be the consequence of muscle mass reduction [36].

Dispersion of muscle strength increases with muscle mass, implying that muscle function is only partially explained by muscle mass. Therefore, although muscle mass constitutes a determinant of muscle strength, we have evidenced the occurrence of dynapenia in our dialysis patients defined as a reduced strength with a maintained muscle mass. This discrepancy between muscle mass and strength has been previously observed in elderly [29] and more recently in CKD [3]. Our study confirms the existence of a dynapenia in CHD patients, and beyond sarcopenia, the term of uremic dynapenia could be used in CHD patients as well.

Our multivariate analysis confirms the link between the major components of PEW and muscle strength, given that MVF reduction was significantly associated with serum albumin, nPCR and CI. (Table 3). The complex relationship between PEW and muscle is further supported by partial correlation analysis (Fig 3). PEW components differentially act on muscle strength and mass. The partial correlation analysis confirms that inflammation and protein intake (estimated by hs-CRP and nPCR respectively) play a pivotal role in serum albumin concentration. While serum albumin acts directly on MVF, inflammation and nPCR are rather determinants of muscle mass which could in turn affect muscle strength.

Physical activity level was reduced in CHD patients, and the Voorrips score of our population is in total agreement with published studies [37]. Here, physical inactivity was independently associated with muscle strength, as previously observed in another cross-sectional study [38]. Given the muscle plasticity, relationship between inactivity and muscle strength could be bidirectional. While muscle atrophy is a cause of physical activity reduction, physical inactivity conversely induces muscle atrophy. Experimental models of disuse (cast, bed rest, etc…) [39] have clearly demonstrated this causal relationship which may probably occur in CHD patients. Indeed, a recent pilot randomized controlled trial has shown significant improvement of muscle volume after resistance-training [40]. A muscle remodelling without muscle fiber atrophy has been reported more especially in CHD patients [41]. Given the clear association between physical inactivity and muscle weakness and specific torque, the potential benefit of exercise training interventions should concern CHD patients with muscle weakness with or without muscle atrophy.

Among variables, PEW (inflammation, undernutrition), physical inactivity and dialysis vintage were the main determinants of dynapenia in CHD patients (Table 4). Inflammation and undernutrition assessed by low albumin levels, were more prevalent in patients with dynapenia as compared to others with muscle wasting. Inflammation could act early in the onset of muscle weakness, as shown in previous studies [3,42]. This supports the hypothesis of a uremic milieu influence that would first impair muscle strength and then reduce muscle mass.

Regarding physical inactivity, models of disuse have shown impairments in the intrinsic cellular muscle contractile properties [39,43]. This may explain why, in elderly subjects, muscle strength declines at a substantially faster rate than muscle mass [29].

A center effect was identified in the univariate analysis. In order to prevent any bias in the statistical analysis, dialysis location was taken into account in the multivariate analysis. However, this location effect is clearly linked to the heterogeneity of dialysis population and comorbidities observed in HD populations. Clearly and as expected, patients from centers 3 and 4, corresponding to self-care dialysis units, presented less PEW and comorbidities than patients from dialysis centers (centers 1 and 2). Since age has been reported to be a major determinant of muscle weakness, it should be postulated that age could be a confusing factor in our study due to the enrollment of elderly patients. However, mean age of our studied population reflects that classically observed in french dialysis centers as reported in the R.E.I.N. French registry (with a mean age of 67 yo when they start chronic hemodialysis)[44]. Our study demonstrated that dynapenia could be frequently observed in CHD patients. It would have been interesting comparing the four groups of patients classified according these two variables. However, due to the relatively small sample size of the study and the initially study design, patients with low MVF but maintained muscle mass could only be compared to all other patients.

The observational study design and the use of investigations at single time points could demonstrate the significant association between muscle strength and nutritional parameters, physical activity and muscle mass but could not support a causal link. However, the strength of this “hypothesis generating” study is to underline that muscle mass is not the only determinant of muscle strength and to highlight the potential role of physical activity in maintaining muscle strength in CHD patients. Experimental studies should provide causal relationship between disturbance of energetic pathway [16,17] and contractile protein dysfunction [45].

## Conclusion

Muscle strength may not be considered as a surrogate for the muscle mass in CHD patients. Beyond atrophy, factors of muscle quality like physical inactivity and PEW conspire towards muscular weakness and specific torque in CHD patients. Further longitudinal studies are needed to assess the relative role of sarcopenia and dynapenia in CHD outcome. In addition, our findings indicate that physical activity could be a promising strategy in addition to nutritional intervention to reduce prevalence of weakness in CHD patients.

## Supporting information

S1 Checklist TREND statement checklist. (DOCX)

S1 Protocol Protocol French version. (DOCX)

S2 Protocol Protocol English version. (DOCX)

## Abbreviations

AIC: Akaike Information criterion
BIA: Bioelectrical impedance analysis
BMI: Body mass index
CI: Creatinine index
CKD: Chronic kidney disease
DXA: Dual X ray absorptiometry
GFR: Glomerular Filtration Rate
HHD: Hand-held dynamometer
hs-CRP: Serum high-sensitivity C-reactive protein
IQR: Interquartile Range
LTM: Lean tissue mass
LTI: Lean tissue index
MVF: Maximal voluntary force
nPCR: Normalised protein catabolism rate
PEW: Protein energy wasting
SMM: Skeletal muscle mass
SMMI: Skeletal muscle mass index
VIF: Variance Inflation Factors

## References

[1] Cruz-Jentoft AJ, Baeyens JP, Bauer JM, Boirie Y, Cederholm T, Landi F, et al Sarcopenia: European consensus on definition and diagnosis: Report of the European Working Group on Sarcopenia in Older People. Age Ageing. 2010;39: 412–423. 10.1093/ageing/afq034 20392703 PMC2886201

[2] Fouque D, Kalantar-Zadeh K, Kopple J, Cano N, Chauveau P, Cuppari L, et al A proposed nomenclature and diagnostic criteria for protein-energy wasting in acute and chronic kidney disease. Kidney Int. 2008;73: 391–398. 10.1038/sj.ki.5002585 18094682

[3] Isoyama N, Qureshi AR, Avesani CM, Lindholm B, Bàràny P, Heimbürger O, et al Comparative associations of muscle mass and muscle strength with mortality in dialysis patients. Clin J Am Soc Nephrol CJASN. 2014;9: 1720–1728. 10.2215/CJN.10261013 25074839 PMC4186520

[4] Kittiskulnam P, Carrero JJ, Chertow GM, Kaysen GA, Delgado C, Johansen KL. Sarcopenia among patients receiving hemodialysis: weighing the evidence. J Cachexia Sarcopenia Muscle. 2017;8: 57–68. 10.1002/jcsm.12130 27897415 PMC5326818

[5] Pereira RA, Cordeiro AC, Avesani CM, Carrero JJ, Lindholm B, Amparo FC, et al Sarcopenia in chronic kidney disease on conservative therapy: prevalence and association with mortality. Nephrol Dial Transplant Off Publ Eur Dial Transpl Assoc—Eur Ren Assoc. 2015;30: 1718–1725. 10.1093/ndt/gfv133 25999376

[6] Chandler JM, Duncan PW, Kochersberger G, Studenski S. Is lower extremity strength gain associated with improvement in physical performance and disability in frail, community-dwelling elders? Arch Phys Med Rehabil. 1998;79: 24–30. 9440412 10.1016/s0003-9993(98)90202-7

[7] Pedruzzi LM, Stockler-Pinto MB, Leite M Jr, Mafra D. Nrf2-keap1 system versus NF-κB: the good and the evil in chronic kidney disease? Biochimie. 2012;94: 2461–2466. 10.1016/j.biochi.2012.07.015 22874821

[8] Stenvinkel P, Heimbürger O, Lindholm B, Kaysen GA, Bergström J. Are there two types of malnutrition in chronic renal failure? Evidence for relationships between malnutrition, inflammation and atherosclerosis (MIA syndrome). Nephrol Dial Transplant Off Publ Eur Dial Transpl Assoc—Eur Ren Assoc. 2000;15: 953–960. 10.1093/ndt/15.7.953 10862630

[9] Carrero JJ, Stenvinkel P, Cuppari L, Ikizler TA, Kalantar-Zadeh K, Kaysen G, et al Etiology of the protein-energy wasting syndrome in chronic kidney disease: a consensus statement from the International Society of Renal Nutrition and Metabolism (ISRNM). J Ren Nutr Off J Counc Ren Nutr Natl Kidney Found. 2013;23: 77–90. 10.1053/j.jrn.2013.01.001 23428357

[10] Obi Y, Qader H, Kovesdy CP, Kalantar-Zadeh K. Latest consensus and update on protein-energy wasting in chronic kidney disease: Curr Opin Clin Nutr Metab Care. 2015;18: 254–262. 10.1097/MCO.0000000000000171 25807354 PMC4506466

[11] Domański M, Ciechanowski K. Sarcopenia: a major challenge in elderly patients with end-stage renal disease. J Aging Res. 2012;2012: 754739 10.1155/2012/754739 22536505 PMC3321443

[12] Francis P, Toomey C, Mc Cormack W, Lyons M, Jakeman P. Measurement of maximal isometric torque and muscle quality of the knee extensors and flexors in healthy 50- to 70-year-old women. Clin Physiol Funct Imaging. 2016; 10.1111/cpf.12332 26749301

[13] Souweine J-S, Boudet A, Chenine L, Leray H, Rodriguez A, Mourad G, et al Standardized Method to Measure Muscle Force at the Bedside in Hemodialysis Patients. J Ren Nutr Off J Counc Ren Nutr Natl Kidney Found. 2017; 10.1053/j.jrn.2017.01.017 28320574

[14] Clark BC, Manini TM. Sarcopenia = / = dynapenia. J Gerontol A Biol Sci Med Sci. 2008;63: 829–834. 18772470 10.1093/gerona/63.8.829

[15] Clark BC, Manini TM. What is dynapenia? Nutr Burbank Los Angel Cty Calif. 2012;28: 495–503. 10.1016/j.nut.2011.12.002 22469110 PMC3571692

[16] Raj DSC, Boivin MA, Dominic EA, Boyd A, Roy PK, Rihani T, et al Haemodialysis induces mitochondrial dysfunction and apoptosis. Eur J Clin Invest. 2007;37: 971–977. 10.1111/j.1365-2362.2007.01886.x 18036031

[17] Gamboa JL, Billings FT, Bojanowski MT, Gilliam LA, Yu C, Roshanravan B, et al Mitochondrial dysfunction and oxidative stress in patients with chronic kidney disease. Physiol Rep. 2016;4: e12780 10.14814/phy2.12780 27162261 PMC4873632

[18] Adey D, Kumar R, McCarthy JT, Nair KS. Reduced synthesis of muscle proteins in chronic renal failure. Am J Physiol Endocrinol Metab. 2000;278: E219–225. 10.1152/ajpendo.2000.278.2.E219 10662705

[19] Canaud B, Granger Vallée A, Molinari N, Chenine L, Leray-Moragues H, Rodriguez A, et al Creatinine index as a surrogate of lean body mass derived from urea Kt/V, pre-dialysis serum levels and anthropometric characteristics of haemodialysis patients. PloS One. 2014;9: e93286 10.1371/journal.pone.0093286 24671212 PMC3966881

[20] Marcelli D, Usvyat LA, Kotanko P, Bayh I, Canaud B, Etter M, et al Body Composition and Survival in Dialysis Patients: Results from an International Cohort Study. Clin J Am Soc Nephrol CJASN. 2015; 10.2215/CJN.08550814 25901091 PMC4491292

[21] Charlson ME, Pompei P, Ales KL, MacKenzie CR. A new method of classifying prognostic comorbidity in longitudinal studies: development and validation. J Chronic Dis. 1987;40: 373–383. 3558716 10.1016/0021-9681(87)90171-8

[22] Daugirdas JT. Second generation logarithmic estimates of single-pool variable volume Kt/V: an analysis of error. J Am Soc Nephrol JASN. 1993;4: 1205–1213. 8305648 10.1681/ASN.V451205

[23] Garred LJ, Barichello DL, Canaud BC, McCready WG. Simple equations for protein catabolic rate determination from pre dialysis and post dialysis blood urea nitrogen. ASAIO J Am Soc Artif Intern Organs 1992. 1995;41: 889–895. 8589472

[24] Janssen I, Heymsfield SB, Baumgartner RN, Ross R. Estimation of skeletal muscle mass by bioelectrical impedance analysis. J Appl Physiol Bethesda Md 1985. 2000;89: 465–471. 10.1152/jappl.2000.89.2.465 10926627

[25] Terrier N, Jaussent I, Dupuy A-M, Morena M, Delcourt C, Chalabi L, et al Creatinine index and transthyretin as additive predictors of mortality in haemodialysis patients. Nephrol Dial Transplant Off Publ Eur Dial Transpl Assoc—Eur Ren Assoc. 2008;23: 345–353. 10.1093/ndt/gfm573 17890748

[26] Maltais F, Decramer M, Casaburi R, Barreiro E, Burelle Y, Debigaré R, et al An official American Thoracic Society/European Respiratory Society statement: update on limb muscle dysfunction in chronic obstructive pulmonary disease. Am J Respir Crit Care Med. 2014;189: e15–62. 10.1164/rccm.201402-0373ST 24787074 PMC4098112

[27] Carrero JJ, Johansen KL, Lindholm B, Stenvinkel P, Cuppari L, Avesani CM. Screening for muscle wasting and dysfunction in patients with chronic kidney disease. Kidney Int. 2016; 10.1016/j.kint.2016.02.025 27157695

[28] Hogrel J-Y, Payan CA, Ollivier G, Tanant V, Attarian S, Couillandre A, et al Development of a French isometric strength normative database for adults using quantitative muscle testing. Arch Phys Med Rehabil. 2007;88: 1289–1297. 10.1016/j.apmr.2007.07.011 17908571

[29] Goodpaster BH, Park SW, Harris TB, Kritchevsky SB, Nevitt M, Schwartz AV, et al The loss of skeletal muscle strength, mass, and quality in older adults: the health, aging and body composition study. J Gerontol A Biol Sci Med Sci. 2006;61: 1059–1064. 17077199 10.1093/gerona/61.10.1059

[30] Voorrips LE, Ravelli AC, Dongelmans PC, Deurenberg P, Van Staveren WA. A physical activity questionnaire for the elderly. Med Sci Sports Exerc. 1991;23: 974–979. 1956274

[31] Anderton N, Giri A, Wei G, Marcus RL, Chen X, Bjordahl T, et al Sedentary Behavior in Individuals With Diabetic Chronic Kidney Disease and Maintenance Hemodialysis. J Ren Nutr. 2015;25: 364–370. 10.1053/j.jrn.2015.01.018 25753603 PMC4469533

[32] Cohen J. Statistical power analysis for the behavioral sciences Rev. ed. New York: Academic Press; 1977.

[33] Baumgartner RN, Ross R, Heymsfield SB. Does adipose tissue influence bioelectric impedance in obese men and women? J Appl Physiol Bethesda Md 1985. 1998;84: 257–262. 10.1152/jappl.1998.84.1.257 9451644

[34] Workeneh BT, Mitch WE. Review of muscle wasting associated with chronic kidney disease. Am J Clin Nutr. 2010;91: 1128S–1132S. 10.3945/ajcn.2010.28608B 20181807

[35] Morishita Y, Kubo K, Haga Y, Miki A, Ishibashi K, Kusano E, et al Skeletal muscle loss is negatively associated with single-pool Kt/V and dialysis duration in hemodialysis patients. Ther Apher Dial Off Peer-Rev J Int Soc Apher Jpn Soc Apher Jpn Soc Dial Ther. 2014;18: 612–617. 10.1111/1744-9987.12174 24674153

[36] Chertow GM, Owen WF, Lazarus JM, Lew NL, Lowrie EG. Exploring the reverse J-shaped curve between urea reduction ratio and mortality. Kidney Int. 1999;56: 1872–1878. 10.1046/j.1523-1755.1999.00734.x 10571796

[37] Zelle DM, Klaassen G, van Adrichem E, Bakker SJL, Corpeleijn E, Navis G. Physical inactivity: a risk factor and target for intervention in renal care. Nat Rev Nephrol. 2017;13: 152–168. 10.1038/nrneph.2016.187 28138130

[38] Baxmann AC, Ahmed MS, Marques NC, Menon VB, Pereira AB, Kirsztajn GM, et al Influence of muscle mass and physical activity on serum and urinary creatinine and serum cystatin C. Clin J Am Soc Nephrol CJASN. 2008;3: 348–354. 10.2215/CJN.02870707 18235143 PMC2390952

[39] Chacon-Cabrera A, Lund-Palau H, Gea J, Barreiro E. Time-Course of Muscle Mass Loss, Damage, and Proteolysis in Gastrocnemius following Unloading and Reloading: Implications in Chronic Diseases. PloS One. 2016;11: e0164951 10.1371/journal.pone.0164951 27792730 PMC5085049

[40] Watson EL, Greening NJ, Viana JL, Aulakh J, Bodicoat DH, Barratt J, et al Progressive Resistance Exercise Training in CKD: A Feasibility Study. Am J Kidney Dis Off J Natl Kidney Found. 2015;66: 249–257. 10.1053/j.ajkd.2014.10.019 25533601

[41] Lewis MI, Fournier M, Wang H, Storer TW, Casaburi R, Cohen AH, et al Metabolic and morphometric profile of muscle fibers in chronic hemodialysis patients. J Appl Physiol Bethesda Md 1985. 2012;112: 72–78. 10.1152/japplphysiol.00556.2011 22016372 PMC3290422

[42] Yardimci B, Sumnu A, Kaya I, Gursu M, Aydin Z, Karadag S, et al Is handgrip strength and key pinch measurement related with biochemical parameters of nutrition in peritoneal dialysis patients? Pak J Med Sci. 2015;31: 941–945. 10.12669/pjms.314.7595 26430434 PMC4590358

[43] Powers SK, Kavazis AN, DeRuisseau KC. Mechanisms of disuse muscle atrophy: role of oxidative stress. Am J Physiol Regul Integr Comp Physiol. 2005;288: R337–344. 10.1152/ajpregu.00469.2004 15637170

[44] Agence de la biomédecine. RAPPORT ANNUEL 2015 Réseau Epidémiologie et Information en Néphrologie. 2015.

[45] Bohé J, Rennie MJ. Muscle protein metabolism during hemodialysis. J Ren Nutr Off J Counc Ren Nutr Natl Kidney Found. 2006;16: 3–16. 10.1053/j.jrn.2005.07.005 16414436

