# Supplementary material for: Physical inactivity and protein energy wasting play independent roles in muscle weakness in maintenance haemodialysis patients
Source: PLoS One. 2018 Aug 1;13(8):e0200061. doi: 10.1371/journal.pone.0200061 (PMC6070183; doi:10.1371/journal.pone.0200061)

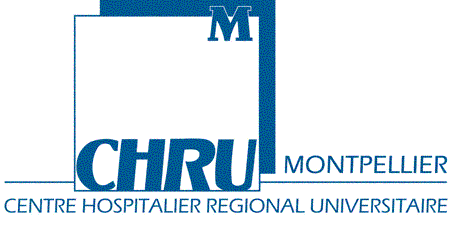


**Protocole de recherche clinique**

Déterminants de la force musculaire en hémodialyse chronique

**Promoteur**

CHRU de Montpellier

Centre Administratif André Bénech

191, avenue du Doyen Gaston Giraud

34295 Montpellier cedex 5

**N° ID RCB : 2015-A01854-45**

**Investigateur coordonnateur**

CRISTOL, Jean-Paul

Spécialité : Biochimie

Statut : PU-PH

CHRU de Montpellier

Hôpital Lapeyronie

Email : jp-cristol@chu-montpellier.fr

Tél : 04 67 33 83 14

Table des matières

[1 Présentation générale 5](#_Toc436151831)

[1.1 Présentation générale de l’étude 5](#_Toc436151832)

[1.2 Principaux correspondants 5](#_Toc436151833)

[1.3 Réseau de recherche - équipes investigatrices et collaboratrices 6](#_Toc436151834)

[1.4 Résumé de l’étude 7](#_Toc436151835)

[2 Justification de l’étude 8](#_Toc436151836)

[3 Objectifs de l’étude 9](#_Toc436151837)

[3.1 Objectif principal 9](#_Toc436151838)

[3.2 Objectifs secondaires 9](#_Toc436151839)

[4 Méthodologie 10](#_Toc436151840)

[4.1 Plan expérimental 10](#_Toc436151841)

[4.2 Sélection des sujets 10](#_Toc436151842)

[4.3 Description de(s) intervention(s) 11](#_Toc436151843)

[4.4 Critères de jugement 11](#_Toc436151844)

[4.5 Paramètres recueillis et méthode de recueil 12](#_Toc436151845)

[4.6 Statistiques : 12](#_Toc436151846)

[5 Faisabilité 13](#_Toc436151847)

[5.1 Potentiel de recrutement 13](#_Toc436151848)

[5.2 Expertises/Disponibilités des équipes 13](#_Toc436151849)

[5.3 Plateau(x) technique(s) spécifique(s) et accessibilité 14](#_Toc436151850)

[5.4 Pré-étude si applicable 14](#_Toc436151851)

[6 Déroulement pratique de l’étude 15](#_Toc436151852)

[6.1 Coordination de l’étude, lieu de réalisation et rôle de chaque équipe 15](#_Toc436151853)

[6.2 Description de la prise en charge et du suivi des sujets 15](#_Toc436151854)

[6.3 Contenu des visites patients 15](#_Toc436151855)

[6.4 Calendrier prévisionnel de l’étude 16](#_Toc436151856)

[7 Collection biologique (si applicable) 17](#_Toc436151857)

[8 Résultats attendus et Perspectives 17](#_Toc436151858)

[9 Protection des personnes 17](#_Toc436151859)

[9.1 Bénéfices et risques prévisibles et connus pour les personnes se prêtant à l’étude 17](#_Toc436151860)

[9.2 Gestion des événements indésirables 17](#_Toc436151861)

[10 Gestion et suivi des données 17](#_Toc436151862)

[10.1 Monitoring et contrôle de qualité 17](#_Toc436151863)

[10.2 Audit et inspection 17](#_Toc436151864)

[10.3 Recueil et gestion des données 17](#_Toc436151865)

[10.4 Mention de la soumission à la CNIL 17](#_Toc436151866)

[10.5 Conservation et archivage 17](#_Toc436151867)

[11 Aspects éthiques et réglementaires 17](#_Toc436151868)

[12 Règles relatives à la publication 17](#_Toc436151869)

[12.1 Rapport d’étude 17](#_Toc436151870)

[12.2 Règles de publication 17](#_Toc436151871)

[12.3 Communication des résultats aux participants 17](#_Toc436151872)

[12.4 Cession des données 17](#_Toc436151873)

[13 Justification des moyens financiers demandés 17](#_Toc436151874)

[14 Références bibliographiques 17](#_Toc436151875)

[14.1 CV des principaux investigateurs 17](#_Toc436151876)

LISTE DES ABREVIATIONS

| FMV | Force maximale volontaire |
| --- | --- |
| BPCO | Bronchopneumopathie chronique obstructive |
| LBM | Lean body mass (masse maigre) |
| BPC | Bonnes Pratiques Cliniques |
| CPP | Comité de Protection des Personnes |
| CNIL | Commission Nationale de l’Informatique et des Libertés |
| CRF | Case Report Form (cahier d’observations) |
| EvIG | Evénement Indésirable Grave |
| EIG | Effet Indésirable Grave |
| EIGI | Effet Indésirable Grave Inattendu |
| ICH | International Conference on Harmonization (Conférence internationale pour l'harmonisation) |
| IDE | Infirmière Diplômée d'Etat |
| INSERM | Institut National de la Santé et de la Recherche Médicale |
| MR | Méthodologie de Référence |
| RCP | Résumé des Caractéristiques d'un Produit |
| SUSAR | Suspected Unexpected Serious Adverse Reaction |
| TEC | Technicien d'Etude Clinique |
| HDC | Hémodialysé chronique |

# Présentation générale

## Présentation générale de l’étude

**Titre de l’étude**

**Déterminants de la force musculaire en hémodialyse chronique**

N° ID-RCB : 2015-A01854-45

Type de recherche : Recherche Biomédicale Hors Produit de Santé (RBM HPS)

Nombre de centre(s) d’inclusions : 4

Nombre total de sujets à inclure : 110

Durée du projet : 1 an

Collection d’échantillons biologiques : ❒ Oui ;    Non

Budget demandé :  **0 €**

Cofinancement : ❒ Oui ;    Non

Si oui, montant et origine du co-financement : Non applicable

## Principaux correspondants

**Promoteur**

CHRU de MONTPELLIER

**Représentant légal du promoteur**

Rodolphe BOURRET

Directeur Général - CHRU de MONTPELLIER

191, avenue du Doyen Gaston Giraud

[dg.secretariat@chu-montpellier.fr](mailto:dg.secretariat@chu-montpellier.fr)

**Contact Direction de la Recherche et Innovation (DRI)**

Anne VERCHERE

Etablissement : CHRU Montpellier - Hôpital La Colombière - Direction Recherche et Innovation

Téléphone : 04 67 33 08 12

[a-verchere@chu-montpellier.fr](mailto:a-verchere@chu-montpellier.fr)

**Coordonnateur (Equipe n°1)**

Jean-Paul CRISTOL

Statut : PU-PH

Spécialité : Biochimie

CHRU de Montpellier

Email : jp-cristol@chu-montpellier.fr

Tél : 04 67 33 83 14

## Réseau de recherche - équipes investigatrices et collaboratrices

| **Titre Prénom NOM** | **Etablissement d'appartenance** | **Service / équipe de rattachement** | **Equipe n°** | **Equipe*** |
| --- | --- | --- | --- | --- |
| Jean-Paul Cristol | CHRU Montpellier Hôp. Lapeyronie | Biochimie / INSERM U1046 | 1 | ⌧ Investigateur coordonnateur |
| Georges Mourad | CHRU Montpellier Hôp. Lapeyronie | Néphrologie | 1 | ⌧ Investigateur associé  ❒ Equipe collaboratrice |
| Maurice Hayot | CHRU Montpellier Hôp. A. Villeneuve | Physiologie respiratoire / INSERM U1046 | 1 | ⌧ Investigateur associé  ❒ Equipe collaboratrice |
| Jacques Mercier | CHRU Montpellier Hôp. A. Villeneuve | Physiologie respiratoire / INSERM U1046 | 1 | ⌧ Investigateur associé  ❒ Equipe collaboratrice |
| Hélène Leray-Moragues | CHRU Montpellier Hôp. Lapeyronie | Néphrologie | 1 | ⌧ Investigateur associé  ❒ Equipe collaboratrice |
| Leila Chenine | CHRU Montpellier Hôp. Lapeyronie | Néphrologie | 1 | ⌧ Investigateur associé  ❒ Equipe collaboratrice |
| Fares Gouzi | CHRU Montpellier Hôp. A. Villeneuve | Physiologie respiratoire / INSERM U1046 | 1 | ⌧ Investigateur associé  ❒ Equipe collaboratrice |
| Nathalie Raynal | AIDER | UAD Montpellier | 2 | ⌧ Investigateur associé  ❒ Equipe collaboratrice |
| Lotfi Chalabi | AIDER | UDM Montpellier | 3 | ⌧ Investigateur associé  ❒ Equipe collaboratrice |
| Laure Patrier | AIDER | UDM Nîmes | 4 | ⌧ Investigateur associé  ❒ Equipe collaboratrice |

**Equipe associée : équipe différente de l’équipe coordonnatrice, dans laquelle figure au moins un investigateur recrutant des patients.*

**Equipe collaboratrice : équipe, qui n’effectue aucun acte direct sur le patient (Ex. : le biologiste, le statisticien…).*

## Résumé de l’étude

**Titre complet du projet**

Déterminants de la force musculaire en hémodialyse chronique

**Contexte et justification**

En hémodialyse chronique, la sarcopénie urémique (avec une prévalence de 20 à 50%), caractérisée par la diminution de la masse et l’altération de la fonction musculaire est un facteur de mortalité important. L’association d’une augmentation de l’inflammation et du stress oxydant, associés à la maladie rénale, définit le syndrome de Malnutrition, Inflammation et Athérogénèse (MIA syndrome). Ce syndrome est impliqué dans le déséquilibre entre anabolisme et catabolisme protéique, ce qui aboutit à une diminution de la concentration de protéines nécessaires à la contraction musculaire. Ainsi la force musculaire, dépendante de la masse musculaire, pourrait être limitée par le MIA syndrome.

**Objectifs principal et secondaires**

- Objectif principal : Déterminer la contribution du MIA syndrome dans la diminution de la force musculaire en hémodialyse chronique.

- Objectifs secondaires : En hémodialyse chronique déterminer :

- La relation entre la force maximale volontaire (FMV) et la masse musculaire.

- La relation entre la FMV et la masse maigre.

- La relation entre la FMV et l’activité physique.

**Méthodes (design étude, population, critères d’inclusion, non inclusion, critères d’évaluation principaux et secondaires, nombre de sujets à inclure, analyse statistique...)**

- Typologie : Etude épidémiologique observationnelle descriptive transversale multicentrique présentant des risques et des contraintes minimes pour le sujet.

- Population (principaux critères d’éligibilité): Patients insuffisants rénaux chroniques hémodialysés chroniques (> 3 mois), âgés de 18 à 90 ans, avec un état clinique stable dans les trois mois précédant l’inclusion.

- Critères de jugement : Le critère de jugement principal est la force maximale volontaire en Newton-mètre.

**Déroulement (nombre de visites, durée des inclusions, durée du suivi, calendrier de l’étude contenu des visites patients, description brève de l’intervention)  :**

Deux visites seront réalisées.

La première visite (pré-inclusion) permettra de vérifier les critères d’inclusion et de non-inclusion, d’informer le patient et d’obtenir sa non opposition à participer à l'étude.

La seconde visite comportera :

- Mesure de la FMV : Avant la séance de dialyse

- Mesure de la masse musculaire par impédancemétrie : après la séance de dialyse

- Recueil des paramètres biologiques nécessaires à l’estimation de la masse musculaire et du MIA syndrome à partir du bilan mensuel correspondant au suivi habituel du patient.

**Résultats attendus et perspectives**

L'identification des déterminants de la force musculaire en hémodialyse chronique permettra de mieux comprendre les facteurs impliqués dans la diminution de la force musculaire. Ainsi cela permettra à terme de proposer des stratégies basées sur le réentrainement musculaire afin de prévenir l'apparition d’un déconditionnement musculaire.

**Mots clés**

Français : Mots clés : hémodialyse, dysfonction musculaire, stress oxydant, inflammation.

# Justification de l’étude

La sarcopénie, définie par l’association d’une perte de masse et de force musculaire, est un processus gériatrique (1). L’existence, en hémodialyse chronique, d’une diminution concomitante de la force et de la masse musculaire définit la sarcopénie urémique, dont la prévalence est de l’ordre de 20 à 50% (2). Dans cette population, la sarcopénie est un marqueur pronostique majeur de mortalité (3).

L’évaluation de la masse musculaire est indispensable en hémodialyse car elle est un reflet de l’activité physique des patients et des apports nutritionnels quotidiens (4). Actuellement deux méthodes, simples et rapides au lit du malade, sont utilisées pour évaluer la masse musculaire des patients hémodialysés chroniques (5,6). La première, l'impédancemétrie, mesurée par le BCM (Body Composition Monitor; Fresenius Medical Care(6)), permet la mesure de la masse maigre (constituée des organes, de la peau, des os et des muscles). La seconde, l'index de créatinine (IC) calculé à partir des paramètres d'hémodialyse et de la production inter-dialytique de créatinine, est un reflet du métabolisme de la créatine donc de la masse musculaire des patients hémodialysés (7). La limite de l’impédancemétrie est la présence d’un excès d’eau extracellulaire inter-dialytique. La mesure nécessite donc d’être standardisée en fonction de la périodicité des séances de dialyse. Le calcul de l’index de créatinine est quant à lui modifié par les variations hebdomadaires d’apports protéiques des patients (7). La force maximale volontaire (FMV) isométrique est corrélée à la surface du muscle (r=0.51) (8) chez les sujets sains, et chez certains patients insuffisants respiratoires chroniques (Bronchopneumopathie chronique obstructive (9)). La mesure de la force musculaire apparait donc comme une nouvelle méthode d’évaluation de la masse musculaire. Ainsi en plus de permettre l’évaluation fonctionnelle du muscle (9), la FMV isométrique du quadriceps permet d’estimer la masse musculaire des patients en dehors de certains paramètres neurologiques(9). En conséquence, chez les patients hémodialysés chroniques, la FMV en association avec l’impédancemétrie et l’index de créatinine permettrait d’avoir une évaluation précise et rapide au lit du malade de l’atrophie musculaire.

La diminution de la masse musculaire, en gériatrie, résulte d'un déséquilibre entre anabolisme et catabolisme protéique (1). L’activation du catabolisme protéique est médiée par le système ubiquitine-protéasome (10). Cette voie est activée par une réduction de l’activité contractile du muscle secondaire à une immobilisation prolongée et par la réduction d’activité physique des patients (11). L’anabolisme est déterminé par l’activité physique et les apports nutritionnels quotidiens. Chez les patients hémodialysés en plus de la présence d’une diminution d’activité, d’une dénutrition et d’une immobilisation plus importante que la population générale, il existe des facteurs spécifiques d’atrophie musculaire (11). En effet l’atrophie musculaire peut être la conséquence d’un stress oxydant accru dans cette population (12) qui aboutit à une activation du système ubiquitine-protéasome (13). Le syndrome de Malnutrition, Inflammation et Athérogénèse (MIA syndrome), défini par une augmentation de l’inflammation et du stress oxydant systémique (14), en induisant un déséquilibre de la balance d’oxydo-réduction (15) pourrait conduire à une diminution de la production de protéines nécessaires à la contraction musculaire (16). Au niveau biologique, les marqueurs du MIA syndrome sont l’augmentation de la CRP et la diminution de l’albumine plasmatique Ainsi l’implication du MIA dans le déséquilibre entre anabolisme et catabolisme protéique, pourrait expliquer son rôle déterminant dans l’apparition et l’entretien d’une atrophie musculaire chez les patients hémodialysés chroniques.

HYPOTHESE DE TRAVAIL : En hémodialyse chronique, le MIA syndrome est un acteur déterminant de la diminution de la masse musculaire estimée par la force maximale volontaire.

# Objectifs de l’étude

## Objectif principal

L’objectif principal de cette étude est de déterminer la contribution du MIA syndrome (augmentation de la CRP et diminution de l’albumine plasmatique) dans la diminution de la FMV chez les patients hémodialysés chroniques.

## Objectifs secondaires

Chez les patients en hémodialyse chronique (HDC) :

- La relation entre la FMV et la masse musculaire appréciée par l'index de créatinine.

- La relation entre la FMV et la masse maigre mesurée par impédancemétrie.

- La corrélation entre la FMV et l’activité physique estimée par le score de Voorrips.

# Méthodologie

## Plan expérimental

### Type d’étude et caractéristiques

Etude épidémiologique observationnelle descriptive transversale multicentrique présentant des risques ou des contraintes minimes pour le sujet.

### Randomisation / appariement

Non applicable

## Sélection des sujets

### Population cible

Patients insuffisants rénaux chroniques au stade ultime, hémodialysés chroniques depuis au moins 3 mois.

### Critères d’inclusion

| Age > à 18 ans et < 90 ans |
| --- |
| Patient insuffisant rénal chronique au stade terminal (DFG< 15ml/min), avec un traitement de suppléance par hémodialyse chronique depuis au moins trois mois. |
| Bilan cardiovasculaire datant de moins de deux ans |
| Etat clinique stable : Dans les trois mois précédant l’inclusion  - Absence d’infection  - Absence de décompensation aigue, de pathologie chronique (poussée d’insuffisance cardiaque, respiratoire, décompensation oedémato-ascitique).  - Absence d’Accident vasculaire cérébral. |
| Etats locomoteurs et neurologiques des membres inférieurs permettant la réalisation des tests fonctionnels proposés. |
| Absence de pathologie musculaire neurodégénérative congénitale ou génétique |
| Recueil de la non opposition à la participation au protocole |
| Obligation d’affiliation ou bénéficiaire d’un régime de sécurité sociale. |

*NB :* la participation du sujet à l’étude est notée dans le dossier médical.

### Critères de non inclusion

| Sujet majeur protégé par la loi ou hors d'état d'exprimer sa non opposition de participation à l'étude selon l'article L 1121-8 du Code de santé publique |
| --- |
| Personne vulnérable selon l'article L.1121-6 du Code de santé publique |
| Sujet non affilié à un régime de sécurité social, ou non bénéficiaire d'un tel régime |
| Insuffisance rénale chronique terminale sans traitement de suppléance |
| Antécédent de transplantation rénale, en cours ou ayant été repris en charge en hémodialyse dans les 365 jours précédant le jour de l'inclusion. |
| Sujet privé de liberté par décision judiciaire ou administrative |
| Contre-indication cardiovasculaire à la pratique d’une activité physique |
| Femmes enceintes ou allaitantes selon l'article L.1121-5 du Code de santé publique |

### Critères de sortie d’étude

Non applicable

## Description de(s) intervention(s)

**Evaluation de la force et de la masse musculaire. :**

La mesure de la force maximale volontaire sera réalisée avant la séance d’hémodialyse

La mesure de la masse musculaire sera réalisée après la séance d’hémodialyse

**Données cliniques :**

A partir des paramètres cliniques et biologiques du patient dans son dossier médical : évaluation de ses antécédents, de ses comorbidités et des paramètres systémiques du MIA syndrome.

## Critères de jugement

### Critère de jugement principal

Chez les patients en HDC, l’analyse multivariée des déterminants de la FMV (en newton par mètre) : Masse musculaire par impédancemetrie et par l’Index de créatinine ; la concentration de CRP et d’albumine plasmatique (MIA syndrome) ; l’activité physique par le score de Voorrips.

### Critères de jugement secondaires

Chez les patients en HDC :

- la corrélation linéaire (Pearson) entre l'index de créatinine et la FMV.

- la corrélation linéaire (Pearson) entre la masse maigre estimée par impédancemetrie et la FMV.

- la corrélation linéaire (Pearson) entre le score de Voorrips et la FMV.

- la corrélation linéaire (Pearson) entre la concentration d’albumine plasmatique et la FMV

- la corrélation linéaire (Pearson) entre la concentration d’hémoglobine et la FMV

## Paramètres recueillis et méthode de recueil

**Paramètres cliniques :**

Questionnaires d’activité physique :

L’évaluation des différents types d’activités (domestiques, de loisirs, sportives) sera faite à l’aide du questionnaire de Baecke modifié par Voorrips, qui informe sur l’activité physique réalisée dans l’année précédente. Ce questionnaire a été utilisé dans plusieurs études antérieures par l'unité INSERM U1046.

Dépistage des comorbidités :

Score de Charlson, déjà validé en néphrologie chez des patients hémodialysés chronique.

**Techniques d'évaluation de la force maximale volontaire isométrique et de la masse musculaire :**

- Microfet2 : Mesure de la force maximale volontaire par un dynamomètre portatif : Microfet-2 ; Hogan Scientific. La mesure se fera en position assise sur un plan dur, sans appui dorsal, sur une table d'examen. L'angulation entre la cuisse et la jambe sera de 90°. La mesure sera réalisée à 2 cm au-dessus de la malléole externe. La longueur du bras de levier est définie par la distance entre les plateaux tibiaux et la zone de fixation de la sangle à la jambe.

- Impédancemétrie bio-analytique : Mesure de la composition corporelle par impédancemétrie multifréquences : Body Composition Monitor (BCM) ; Fresenius Medical Care. Le patient sera en position allongé au repos depuis au moins 15 minutes après la fin de la séance d’hémodialyse.

- Index de créatinine : Mesure de la production quotidienne de créatinine par l'index de créatinine estimé à partir des données anthropométriques (âge, sexe), de la dose de dialyse (Kt/V urée) et du taux de créatinine pré-dialytique. L'index de créatinine dérive de la modélisation de la cinétique de la créatinine (10). Ce modèle est reconnu comme étant le reflet de la masse musculaire et du statut nutritionnel des patients en HDC (10).

- Techniques de dosage des paramètres systémiques du MIA syndrome :

Dosage de la créatinine de l’albumine et de la CRP : respectivement par méthode enzymatique, immunonéphélométrie et immunoturbidimétrie.

## Statistiques :

### Nombre prévu de personnes à inclure dans l’étude

En considérant un modèle linéaire multivarié à 5 covariables (albumine – CRP – Score de Voorrips – LBM – Index de créatinine), un risque de première espèce de 5 %, une puissance souhaitée de 90% et une force de l'effet de 0,15 comme proposé par Cohen (Cohen behavioral sciences 1988) il sera nécessaire d'inclure 110 patients. Le calcul a été effectué sur le logiciel R 3.1.1 (R Coreteam, Vienne, Autriche) package pwr.

### Description des méthodes statistiques prévues, y compris du calendrier des analyses intermédiaires prévues

- Analyse descriptive et comparabilité des groupes:

Une analyse descriptive initiale sera réalisée. Pour les variables qualitatives, cette description comprendra l'effectif. Concernant les variables quantitatives, la description comprendra l'effectif, la moyenne, l'écart type, la médiane ainsi que les valeurs extrêmes selon la distribution.

- Analyse des critères de jugements:

Les critères de jugement seront comparés à l’aide d’un test de Student. Si les conditions d'applications du test de Student ne sont pas respectées (distribution normale, égalité des variances), un test de Mann-Whitney sera utilisé pour comparer ces 2 groupes.

Les corrélations univariées entre les critères de jugement seront recherchées à l’aide du test de Pearson ou Spearman, en fonction du caractère normal ou non de la distribution des variables. L’analyse multivariée se fera par régression linéaire multiple.

Un test sera considéré comme significatif lorsque son degré de signification p est inférieur au seuil de significativité de 5 %.

Aucune analyse intermédiaire n'étant prévue, un test sera considéré comme significatif lorsque son degré de signification p est inférieur au seuil de significativité de 5 %. Les analyses seront effectuées à l’aide du logiciel R version 2.13.0 après le verrouillage de la base de données et l’approbation du Plan d’Analyses Statistiques.

# Faisabilité

## Potentiel de recrutement

Le nombre de sujets susceptibles d’être inclus est de 200 malades. En effet, le nombre de patients hémodialysés dans les quatre centres investigateurs est de 200 malades. Le taux prévisible de patients ne répondant pas aux critères d’inclusion et de non inclusion est de 30 %. Le taux prévisible de refus de participer au protocole est de 15 %. Soit au total un nombre d’inclusion possible de 110 sujets hémodialysés chroniques.

## Expertises/Disponibilités des équipes

**La coordination de l'essai** : (comité de pilotage, chef de projet) sera assurée par le Pr JP Cristol, investigateur principal de l’étude et chef de service du Département de biochimie du CHU, en collaboration avec le Pr M. Hayot, responsable du Département de Physiologie Clinique du CHU, et le Pr J. Mercier, responsable de l’équipe de recherche INSERM U-1046.

**Le rôle de chaque équipe sera attribué avant l’étude :**

-Le recrutement sera réalisé par l’ensemble des médecins investigateurs des équipes CHRU/AIDER.

Equipe 1 : Site CHU GCS Montpellier : Responsable Pr JP Cristol

Equipe 2 : Site AIDER Valsiere : Responsable Dr N Raynal

Equipe 3 : Site AIDER Montpellier : Responsable Dr L Chalabi

Equipe 4 : Site AIDER Nîmes : Responsable Dr L Patrier

- L’inclusion des patients ainsi que le recueil, la saisie et le traitement des données seront assurés par les médecins investigateurs des équipes CHRU/AIDER dans chaque centre.

- Les évaluations fonctionnelles et cliniques finales seront assurées par l’équipe du département de physiologie CHRU/INSERM.

- Le contrôle de qualité des analyses biologiques sera effectué par le service de biochimie.

- A l’initiative du médecin coordonnateur, une réunion de mise en place de l’étude sera programmée au début de l’étude ainsi que des réunions régulières de suivi.

## Plateau(x) technique(s) spécifique(s) et accessibilité

Equipe 1 : Centre de dialyse chronique : GCS Unité de dialyse médicalisé (UDM) de Lapeyronie

Adresse : 371, avenue doyen Gaston Giraud, 34295 Montpellier cedex 5

Equipe 2 : Centre de dialyse chronique : AIDER Unité d’autodialyse (UAD) de la Valsiere

Adresse : 787 Rue de la Valsière, 34790 Grabels

Equipe 3 : Centre de dialyse chronique : AIDER Unité de dialyse médicalisée (UDM) de Lapeyronie :

Adresse : 371, avenue doyen Gaston Giraud, 34295 Montpellier cedex 5

Equipe 4 : Centre de dialyse chronique : AIDER Unité de dialyse médicalisée (UDM) de Nîmes :

Adresse : Rue du Professeur Robert Debré, 30000 Nîmes

## Pré-étude si applicable

Non applicable

# Déroulement pratique de l’étude

## Coordination de l’étude, lieu de réalisation et rôle de chaque équipe

**Lieux de réalisation des inclusions et des évaluations musculaires :**

Le recrutement et l’inclusion des patients se feront dans 4 centres d’hémodialyse chronique du Languedoc Roussillon. Les centres investigateurs sont les suivants :

Centre de dialyse chronique : AIDER Unité d’autodialyse (UAD) de la Valsiere

Adresse : 787 Rue de la Valsière, 34790 Grabels

Centre de dialyse chronique : AIDER Unité de dialyse médicalisée (UDM) de Lapeyronie :

Adresse : 371, avenue doyen Gaston Giraud, 34295 Montpellier cedex 5

Centre de dialyse chronique : AIDER Unité de dialyse médicalisée (UDM) de Nîmes :

Adresse : Rue du Professeur Robert Debré, 30000 Nîmes

Centre de dialyse chronique : GCS Unité de dialyse médicalisé (UDM) de Lapeyronie

Adresse : 371, avenue doyen Gaston Giraud, 34295 Montpellier cedex 5

## Description de la prise en charge et du suivi des sujets

### Modalités pratiques de recrutement

Aucun examen ne se fera en dehors de ces 4 centres investigateurs. Chaque patient sera inclus dans son centre d’hémodialyse habituel. Pour chaque patient, un des médecins investigateurs vérifiera si le patient répond à l’ensemble des critères d’inclusion et de non-inclusion.

### Modalités pratiques d’information et de recueil de la non opposition à la participation à l’étude

L’inclusion des patients hémodialysés chroniques se fera pendant la séance de dialyse. Les patients seront informés par un des investigateurs au préalable par une note d’information et une explication orale. Cette visite d’information correspondra à la visite de pré-inclusion V1.

Après une période de réflexion d’une semaine, si le patient accepte de participer, le médecin investigateur recueillera son consentement éclairé écrit et signé et le patient sera inclus. Cette seconde visite correspondra à la visite V2.

## Contenu des visites patients

**Visite V1:** Vérification des critères d'inclusion et de non inclusion, information du patient, examen clinique.

**Visite V2:**

Recueil du consentement éclairé écrit et signé par le sujet participant au protocole et par un des investigateurs de l’étude. (Un original sera conservé par l'investigateur, un original sera remis au participant à la recherche.)

Avant la séance d’hémodialyse : mesure de la force maximale volontaire.

Après la séance d’hémodialyse : mesure de la masse maigre.

**Remarque :**

- La mesure de la force maximale volontaire de chaque patient est réalisée avant la séance de dialyse pour éviter toute fatigue supplémentaire secondaire à la dialyse. Elle ne retardera pas la prise en charge du patient, puisque elle sera réalisée pendant la préparation du poste de dialyse par le personnel soignant (montage et désinfection de la machine). L’évaluation de la force musculaire est actuellement recommandée en hémodialyse chronique car nécessaire au dépistage de la sarcopénie urémique.

- La mesure de la masse maigre par impédancemetrie se fera 15 minutes après la fin de la séance de dialyse du patient, afin de permettre un retour à l’équilibre entre les différents secteurs liquidiens (interstitielle – vasculaire – intracellulaire) (6). Afin de ne pas retarder le retour à domicile du patient la mesure sera réalisée immédiatement après l’arrêt de la circulation extracorporelle (CEC) et fermeture de l’abord vasculaire (1/ Si Fistule artério-veineuse : compression manuelle 2/ Si cathéters dialyse : fermeture et pansement occlusif). L’utilisation d’un impédancemetre en hémodialyse chronique est recommandée car c’est un outil nécessaire d’une part à l’estimation du poids sec en hémodialyse chronique et d’autre part au dépistage de la sarcopénie urémique.

- Les paramètres biologiques nécessaires au calcul de l’index de créatinine et à l’estimation du MIA syndrome seront recueillis à partir du bilan mensuel du mois où ont été réalisées les mesures de la force maximale volontaire et de la masse maigre de chaque patient.

**NB :**

Les paramètres biologiques sanguins analysés dans le cadre de cette étude font partie du bilan biologique mensuel (ou semestriel) correspondant au suivi habituel du patient hémodialysé et ne nécessitent donc pas de prélèvement sanguin supplémentaire.

*Calendrier des évaluations*

| **Calendrier des évaluations** | **Sélection, inclusion V1** | **Visite de suivi V2** |
| --- | --- | --- |
| Information du sujet | **x** |  |
| Vérification des CI / CNI | **x** |  |
| Examen clinique | **x** |  |
| Recueil du consentement éclairé écrit et signé |  | **x** |
| Evaluation de la force musculaire maximale volontaire |  | **x** |
| Evaluation de la masse maigre |  | **x** |
| Questionnaire d’activité physique, anxiété, dépression, fatigue |  | **x** |

## Calendrier prévisionnel de l’étude

Obtention des avis règlementaires : 3 mois (M0 – M3)

Période d’inclusion : 6 mois

Durée du Suivi : Non applicable

Fin du suivi : Non applicable

Durée du gel de la base de données et des analyses statistiques/valorisation : 2 mois

Rédaction du rapport final : 2 mois

Date de communication du rapport final : Décembre 2016

Publication : Janvier 2017

Durée prévisionnelle de l’étude : 13 mois (de M0 à M13)

# Collection biologique (si applicable)

Non applicable

# Résultats attendus et Perspectives

**ORIGINALITE ET CARACTERE INNOVANT :**

L'originalité de ce projet est d’étudier conjointement la masse et la force musculaire des patients en hémodialyse chronique (HDC). Premièrement l’évaluation de la force musculaire repose sur un outil de dépistage simple, rapide, et non invasif qui permettra d’établir des valeurs de FMV en fonction de l'âge, du poids, de la taille et du sexe des patients en hémodialyse chronique. Deuxièmement l’évaluation de la masse musculaire, délicate en hémodialyse à cause de l’excès d’eau extracellulaire se fera par deux méthodes complémentaires, par impédancemetrie et par l’index de créatinine.

Chez le patient hémodialysé chronique, la dysfonction musculaire constitue un facteur de morbi-mortalité et d'altération de la qualité de vie importante (17). Peu d'études se sont intéressées à la caractérisation de la dysfonction musculaire et aux déterminants à l'origine de ces anomalies cliniques. On observe chez ces patients à la fois une diminution de la masse musculaire estimée par le lean body mass (LBM) (18), et une altération de la fonction musculaire (19) conduisant à une diminution de la capacité à réaliser un effort (4). Le syndrome inflammatoire et le stress oxydant dans le cadre du syndrome de malnutrition-inflammation-athérogénèse (MIA) (20) sont deux acteurs importants de ce dysfonctionnement musculaire (19)(21).

**PERSPECTIVES :**

Cohorte prospective de patients hémodialysés chroniques : Evaluer, par un suivi prospectif, la survie des patients hémodialysés chroniques en fonction de leur force et de leur masse musculaire.

Réentrainement à l’effort des patients en hémodialyse chronique : Evaluer après un reconditionnement musculaire la force et la masse musculaire des patients en hémodialyse chronique.

# Protection des personnes

## Bénéfices et risques prévisibles et connus pour les personnes se prêtant à l’étude

### Bénéfices

**BENEFICES ATTENDUS POUR LE PATIENT ET/OU POUR LA SANTE PUBLIQUE :**

Identifier avec précision les déterminants de la force musculaire en HDC permettra de mieux comprendre les facteurs impliqués dans la diminution de la force musculaire des patients hémodialysés chroniques.

Enfin, la meilleure compréhension des mécanismes de la dysfonction musculaire nous permettra de proposer des stratégies préventives précoces basées sur le réentrainement musculaire afin de prévenir l'apparition d’un déconditionnement musculaire.

### Risques

Les risques que présente la recherche biomedicale :

1/ Utilisation du Microfet2 : Risque de crampes musculaires: L’évaluation de la force maximale par le Microfet2 peut s'accompagner de crampes musculaires. Toutefois, ce risque est limité en raison de la rapidité de la mesure. Par ailleurs, cette mesure est effectuée avant la séance de dialyse, ce qui évite une majoration des crampes secondaires à la dialyse.

2/ Utilisation d’un impédancemètre : La mesure de la composition corporelle par impédancemétrie est réalisée 15 min après le débranchement du patient, ce qui suppose un retour au domicile retardé et un fardeau de la maladie rénale alourdi. Or, après le débranchement, le patient est tenu de rester allongé durant une période incompressible (en moyenne 15-20 minutes), le temps que le personnel soignant s'assure de la bonne fermeture de l’abord vasculaire et mesure certaines constantes (fréquence cardiaque, tension artérielle). La mesure de la composition corporelle est prévue durant cette période incompressible, le retour au domicile n'en sera donc pas retardé.

## Gestion des événements indésirables

La recherche sera conduite dans le respect de la réglementation française en vigueur, notamment les dispositions relatives à la recherche biomédicale : Directive Européenne 2001/20/CE, Loi de santé publique du 9 août 2004, Décret d’application n°2006-477 du 26 avril 2006.

Concernant la vigilance du projet, les responsabilités de l’investigateur et du promoteur, la déclaration des évènements indésirables graves, les rapports annuels de sécurité seront suivis et réalisés en accord avec la règlementation.

***Définitions***

Evènement indésirable (EI) : Toute manifestation nocive survenant chez une personne qui se prête à une recherche biomédicale que cette manifestation soit liée ou non à la recherche ou au produit sur lequel porte cette recherche.

Cette définition s'applique à :

Toute exacerbation d'une maladie préexistante,

Toute augmentation de la fréquence ou de la sévérité d'un événement / état clinique intermittent préexistant, tout symptôme / maladie découvert après le début de l'étude, même s'il existait avant l'inclusion du sujet dans l'essai.

Tout symptôme / maladie présent à l'inclusion du sujet et s'aggravant pendant l'essai.

**Effet indésirable (EfI)** : toute réaction nocive et non désirée imputable à un ou plusieurs paramètres caractéristiques du protocole de recherche (procédures, méthodes, actes pratiqués ou produits faisant l'objet de la recherche ou utilisés pour les besoins de la recherche).

Pour chaque évènement indésirable, l'investigateur et le promoteur évaluent son lien de causalité avec la recherche. Tout évènement indésirable considéré par l'investigateur comme ayant un lien de causalité scientifiquement raisonnable avec la recherche est qualifié d'effet indésirable. L'expression "lien de causalité scientifiquement raisonnable" signifie généralement qu'il existe une preuve ou un argument permettant de suggérer, sur le plan scientifique, une relation de cause à effet entre la réaction nocive et non désirée observée et la recherche.

**Effets indésirable inattendu (EfI-I)** : tout effet indésirable dont la nature, la sévérité ou l'évolution ne concorde pas avec les informations figurant dans le document de référence (Résumé des Caractéristiques du Produits, Brochure Investigateur…ou notice d'utilisation du DM)

**Faits nouveaux** : Toute nouvelle donnée de sécurité, pouvant conduire à une réévaluation du rapport des bénéfices et des risques de la recherche ou du médicament expérimental, ou qui pourrait être suffisant pour envisager des modifications dans l'administration du médicament expérimental, dans la conduite de la recherche.

**Evènement ou effet indésirable grave (EIG):** Un événement ou effet indésirable grave est un événement (ou effet) :

- dont l'évolution est fatale,

- qui met en danger la vie de la personne qui se prête à la recherche,

- qui entraîne une incapacité ou un handicap important ou durable,

- qui provoque une hospitalisation ou une prolongation d'hospitalisation

- qui a pour conséquence une anomalie ou une malformation congénitale

- tout autre événement ne répondant pas aux qualifications énumérées ci-dessus, mais pouvant être considéré comme « potentiellement grave » notamment certaines anomalies biologiques

- événement médicalement pertinent selon le jugement de l’investigateur

- un événement nécessitant une intervention médicale pour prévenir l’évolution vers un des états précités.

Par exemple, ces événements peuvent être un traitement intensif aux urgences hospitalières ou au domicile du participant à la recherche pour un bronchospasme allergique, une crise convulsive ou des troubles de la coagulation.

L'expression " mettre la vie en danger" est réservée à une menace vitale immédiate, au moment de l'événement indésirable, et ce, indépendamment des conséquences qu'aurait une thérapeutique correctrice ou palliative.

Certaines circonstances nécessitant une hospitalisation ne relèvent pas du critère de gravité « hospitalisation/prolongation d’hospitalisation » comme :

- admission pour raisons sociale ou administrative

- hospitalisation prédéfinie par le protocole

- hospitalisation pour traitement médical ou chirurgical programmé avant la recherche

- passage en hôpital de jour

**Evènements graves à ne pas déclarer immédiatement**

Recenser ici les évènements indésirables graves ne nécessitant pas une déclaration immédiate en accord avec les autorités de santé.

**Listing des effets indésirables attendus avec ce protocole**

**Responsabilités de l'investigateur**

*Modalités de recueil des évènements indésirables*

Tous les évènements indésirables seront notés sur les formulaires de recueil des évènements indésirables du cahier d'observation. Chaque évènement indésirable observé sera consigné individuellement. Tous les évènements indésirables doivent être gradés en intensité.

*Déclaration des EIG*

L'investigateur évalue chaque évènement indésirable au regard de sa gravité.

L’investigateur doit notifier au promoteur, sans délai, à compter du jour où il en a connaissance, tous les évènements indésirables graves survenus dans l'essai, à l'exception de ceux qui sont recensés dans le protocole comme ne nécessitant pas une notification immédiate.

Cette notification initiale se fait par fax à l'adresse suivante :

Direction de la recherche et de l'Innovation

Pharmacovigilance des Essais Cliniques

04 67 33 91 72

Cette notification initiale fait l'objet d'un rapport écrit et doit être suivie si nécessaire par un ou des rapport(s) complémentaire(s) écrit(s) détaillé(s).

*Evaluation de la causalité :*

L'investigateur doit évaluer le lien de causalité des évènements avec la recherche (médicament expérimental, comparateur, technique chirurgical…). Le lien de causalité est binaire (relié/non relié).

**Responsabilités du promoteur**

*Déclarations des EIGI et des faits nouveaux*

Le promoteur doit évaluer le lien de causalité entre l'évènement indésirable grave et la recherche. Il évalue si l'effet indésirable est attendu ou inattendu en s'aidant du document de référence.

Il déclare dans les délais réglementaires tous les faits nouveaux et les effets indésirables graves et inattendus à l'EMA (saisie EudraVigilance, base de données de pharmacovigilance Européenne), aux autorités de santé compétentes et aux comités d'éthique concernés et informe les investigateurs.

La déclaration réglementaire est faite dans un délai maximum de :

- 7 jours calendaires pour les effets indésirables graves inattendus fatals ou menaçant le pronostic vital. Dans ce cas, des informations complémentaires pertinentes doivent être recherchés et transmises dans un nouveau délai de 8 jours.

- 15 jours calendaires pour tous les autres effets graves inattendus. De même des informations complémentaires pertinentes doivent être recherchées et transmises dans un nouveau délai de 8 jours.

*Rapport annuel de sécurité*

A la date anniversaire de l'autorisation d'essai délivrée par les Autorités de santé dans le cadre des essais portant sur un produit de santé, ou à la date anniversaire de la première inclusion dans les autres recherches biomédicales, le promoteur rédige un rapport annuel de sécurité comprenant :

- la liste des effets indésirables graves susceptibles d'être lié(s) à la recherche incluant les effets graves inattendus et attendus.

- une analyse concise et critique de la sécurité des patients se prêtant à la recherche.

Ce rapport peut être soumis à l'investigateur coordonnateur pour approbation.

Ce rapport est envoyé aux autorités compétentes et aux comités d'étiques concernés dans les 60 jours suivant la date anniversaire.

*Comité de surveillance indépendant* (si non mis en place, mettre si dessous les justifications)

Un comité indépendant de surveillance de l'essai sera constitué dans le cadre de cette étude. Il s'agit d'un comité consultatif chargé de garantir la protection des patients, de s'assurer que l'essai est conduit de façon éthique, d'évaluer le rapport bénéfice/risque de l’essai et d’assurer la revue indépendante des résultats scientifiques en cours ou à la fin de l’essai.

Ses membres, compétents dans le domaine des essais cliniques (pathologie, méthodologie…), ne sont pas impliqués dans l’étude. Le choix des membres du Comité de Surveillance Indépendant est fait par le promoteur en collaboration avec l’investigateur coordonnateur.

Ils sont nommés et mandatés par le promoteur pour la durée de l’étude. Ils s’engagent sur leur participation à titre bénévole comme sur le respect de la confidentialité des données.

Le Comité de Surveillance Indépendant reçoit les versions successives du protocole, les Rapports annuels de Sécurité. Il peut être sollicité à tout moment par le promoteur si un effet indésirable grave inattendu ou un événement indésirable grave présente une difficulté particulière d’analyse, si des données susceptibles de modifier le rapport bénéfice/risque apparaissent en cours d’étude.

Le Comité de Surveillance Indépendant analyse les données qui lui sont transmises, peut demander des compléments d'informations. Il émet un avis et des recommandations quant au devenir de l’étude (poursuite, amendements, arrêt…).

Il sera constitué de personnes extérieures à la recherche dont nécessairement :

- un clinicien spécialiste de la pathologie étudiée,

- un pharmacologue/pharmaco vigilant

- un méthodologiste/biostatisticien.

La composition et les règles de fonctionnement (rythme des réunions…) sont définies dans la charte du Comité de surveillance Indépendant.

# Gestion et suivi des données

## Monitoring et contrôle de qualité

Le monitoring du projet sera assuré par un assistant de recherche clinique délégué par le promoteur (ARC promoteur). Un monitoring adapté sera mis en œuvre en fonction d’une grille de risques liés au projet. Conformément à cette grille, l’ARC promoteur réalisera des visites régulières des centres d'investigation du projet (visite de mise en place, de suivi selon le rythme des inclusions et une visite de fermeture). Toute visite fera l’objet d’un rapport de monitorage par compte-rendu écrit (traçabilité des visites).

## Audit et inspection

Les investigateurs acceptent de se conformer aux exigences du promoteur et de l’Autorité Compétente en ce qui concerne un audit ou une inspection de la recherche.

L’audit pourra s’appliquer à tous les stades de la recherche, du développement du protocole à la publication des résultats et au classement des données utilisées ou produites dans le cadre de la recherche.

## Recueil et gestion des données

Le participant ne sera identifié que par un numéro d’identification unique, la première lettre du nom, la première lettre du prénom et l’année de naissance. Une liste d'identification des sujets sera conservée dans le dossier de l’investigateur. L’investigateur s’assurera que l’anonymat de chaque personne participant à l’étude est garanti. Les informations seront recueillies pour chaque participant sur un cahier d’observation standardisé rempli par l’investigateur ou le coinvestigateur.

Documents sources : Les documents sources sont les documents originaux, les données et les dossiers, à partir desquels les données concernant les participants à la recherche sont reportées dans le cahier d’observation. L’investigateur s’engage à autoriser un accès direct aux données sources de l’étude lors des visites de contrôle, d’audit ou d’inspection.

## Mention de la soumission à la CNIL

Les informations recueillies lors de cette étude pourront faire l'objet d'un traitement informatique. Le fichier sera réalisé en conformité avec la procédure MR001 de la CNIL (Commission Nationale de l'Informatique et des Libertés) applicable.Commission Nationale de l’Informatique et des Libertés).

## Conservation et archivage

La clôture de l’essai incluant la fermeture des centres sera effectuée en accord avec les Bonnes Pratiques Cliniques et ICH. Les dossiers médicaux, administratif, et les cahiers d'observations seront conservés pendant toute la durée de l'étude dans le service, puis archivés pendant une durée minimum de 15 ans.

# Aspects éthiques et réglementaires

La recherche sera conduite dans le respect de la réglementation française en vigueur, notamment les dispositions relatives à la recherche biomédicale du Code de la Santé publique, articles L1121-1 et suivants (loi de santé publique du 9 août 2004), les lois de Bioéthiques, la loi informatique et libertés, la déclaration d’Helsinki, et les Bonnes Pratiques Cliniques.

**Comité de Protection des personnes (CPP) :**

Avant la mise en œuvre de la recherche, le promoteur soumettra le projet à l’avis du Comité de Protection des Personnes Sud Méditerranée I. **(Dans le cadre de RBM)**

**Autorité compétente (ANSM):**

Avant de réaliser ou de faire réaliser une recherche biomédicale, le promoteur de cette recherche adresse une demande d'autorisation à l'autorité compétente. **(Dans le cadre de RBM)**

**Information et consentement des participants :**

Préalablement à la réalisation d'une rechercher biomédicale sur une personne, le consentement libre, éclairé et écrit du sujet doit être recueilli après qu'il ait été informé, par l'investigateur lors d’une visite préalable (V1) et un délai de réflexion suffisant de une semaine.

L'information destinée aux participants de l'essai doit comprendre l'ensemble des éléments définis dans la loi de santé publique du 9 août 2004 et doit être écrite de façon simple, dans un langage compréhensible par (les parents et) le participant. Après en avoir pris connaissance, le formulaire de consentement doit être daté et signé personnellement par le participant à la recherche et l'investigateur (un original sera conservé par l'investigateur, un original sera remis au participant à la recherche).

**Assurance :** Le CHU de Montpellier, promoteur de l’étude, souscrit pour toute la durée de l'étude une assurance garantissant sa propre responsabilité civile ainsi que celle de tout intervenant impliqué dans la réalisation de l'essai, indépendamment de la nature des liens existant entre les intervenants et le promoteur. **(Dans le cadre de RBM)**.

# Règles relatives à la publication

## Rapport d’étude

Un rapport final daté et signé par l'investigateur sera transmis au promoteur, qui le transmettra aux autorités compétentes dans les 12 mois suivant la fin de l’étude.

## Règles de publication

Toute communication écrite ou orale des résultats de la recherche doit recevoir l’accord préalable de l’investigateur coordonnateur et du promoteur.

Le CHU de Montpellier est propriétaire des données et aucune utilisation ou transmission à un tiers ne peut être effectuée sans son accord préalable.

Le CHU de Montpellier, promoteur de la recherche, doit être mentionné dans les publications selon le modèle d’écriture d’adresse suivant:

CHU Montpellier, département /service, ville, F-code postal, pays.

## Communication des résultats aux participants

Conformément à la loi n°2002-303 du 4 mars 2002, les sujets sont informés, à leur demande, des résultats globaux de la recherche par l’investigateur.

## Cession des données

Les conditions de cession de tout ou partie de la base de données de la recherche sont décidées par le promoteur de la recherche et font l’objet d’un contrat écrit.

# Justification des moyens financiers demandés

Non applicable

# Références bibliographiques

1. Cruz-Jentoft AJ, Baeyens JP, Bauer JM, Boirie Y, Cederholm T, Landi F, et al. Sarcopenia: European consensus on definition and diagnosis: Report of the European Working Group on Sarcopenia in Older People. Age Ageing. 2010 Jul;39(4):412–23.

2. Isoyama N, Qureshi AR, Avesani CM, Lindholm B, Bàràny P, Heimbürger O, et al. Comparative associations of muscle mass and muscle strength with mortality in dialysis patients. Clin J Am Soc Nephrol. 2014 Oct 7;9(10):1720–8.

3. Pereira RA, Cordeiro AC, Avesani CM, Carrero JJ, Lindholm B, Amparo FC, et al. Sarcopenia in chronic kidney disease on conservative therapy: prevalence and association with mortality. Nephrol Dial Transplant. 2015 Oct;30(10):1718–25.

4. Domański M, Ciechanowski K. Sarcopenia: a major challenge in elderly patients with end-stage renal disease. J Aging Res. 2012;2012:754739.

5. Terrier N, Jaussent I, Dupuy A-M, Morena M, Delcourt C, Chalabi L, et al. Creatinine index and transthyretin as additive predictors of mortality in haemodialysis patients. Nephrol Dial Transplant. 2008 Jan;23(1):345–53.

6. Marcelli D, Usvyat LA, Kotanko P, Bayh I, Canaud B, Etter M, et al. Body Composition and Survival in Dialysis Patients: Results from an International Cohort Study. Clin J Am Soc Nephrol. 2015 Apr 21;

7. Canaud B, Granger Vallée A, Molinari N, Chenine L, Leray-Moragues H, Rodriguez A, et al. Creatinine index as a surrogate of lean body mass derived from urea Kt/V, pre-dialysis serum levels and anthropometric characteristics of haemodialysis patients. PLoS ONE. 2014;9(3):e93286.

8. Maughan RJ, Watson JS, Weir J. Strength and cross-sectional area of human skeletal muscle. J Physiol (Lond). 1983 May;338:37–49.

9. Seymour JM, Spruit MA, Hopkinson NS, Natanek SA, Man WD-C, Jackson A, et al. The prevalence of quadriceps weakness in COPD and the relationship with disease severity. Eur Respir J. 2010 Jul;36(1):81–8.

10. Workeneh BT, Mitch WE. Review of muscle wasting associated with chronic kidney disease. Am J Clin Nutr. 2010 Apr;91(4):1128S – 1132S.

11. Panaye M, Kolko-Labadens A, Lasseur C, Paillasseur J-L, Guillodo MP, Levannier M, et al. Phenotypes influencing low physical activity in maintenance dialysis. J Ren Nutr. 2015 Jan;25(1):31–9.

12. Baptista G, Dupuy A-M, Jaussent A, Durant R, Ventura E, Sauguet P, et al. Low-grade chronic inflammation and superoxide anion production by NADPH oxidase are the main determinants of physical frailty in older adults. Free Radic Res. 2012 Sep;46(9):1108–14.

13. Rajan VR, Mitch WE. Muscle wasting in chronic kidney disease: the role of the ubiquitin proteasome system and its clinical impact. Pediatr Nephrol. 2008 Apr;23(4):527–35.

14. Stenvinkel P, Heimbürger O, Lindholm B, Kaysen GA, Bergström J. Are there two types of malnutrition in chronic renal failure? Evidence for relationships between malnutrition, inflammation and atherosclerosis (MIA syndrome). Nephrol Dial Transplant. 2000 Jul;15(7):953–60.

15. Yazdi PG, Moradi H, Yang J-Y, Wang PH, Vaziri ND. Skeletal muscle mitochondrial depletion and dysfunction in chronic kidney disease. Int J Clin Exp Med. 2013;6(7):532–9.

16. Adey D, Kumar R, McCarthy JT, Nair KS. Reduced synthesis of muscle proteins in chronic renal failure. Am J Physiol Endocrinol Metab. 2000 Feb;278(2):E219–25.

17. Noori N, Kopple JD, Kovesdy CP, Feroze U, Sim JJ, Murali SB, et al. Mid-arm muscle circumference and quality of life and survival in maintenance hemodialysis patients. Clin J Am Soc Nephrol. 2010 Dec;5(12):2258–68.

18. Terrier N, Senécal L, Dupuy A-M, Jaussent I, Delcourt C, Leray H, et al. Association between novel indices of malnutrition-inflammation complex syndrome and cardiovascular disease in hemodialysis patients. Hemodial Int. 2005 Apr;9(2):159–68.

19. Fahal IH, Bell GM, Bone JM, Edwards RH. Physiological abnormalities of skeletal muscle in dialysis patients. Nephrol Dial Transplant. 1997 Jan;12(1):119–27.

20. Desmeules S, Lévesque R, Jaussent I, Leray-Moragues H, Chalabi L, Canaud B. Creatinine index and lean body mass are excellent predictors of long-term survival in haemodiafiltration patients. Nephrol Dial Transplant. 2004 May;19(5):1182–9.

21. Castaneda C, Gordon PL, Parker RC, Uhlin KL, Roubenoff R, Levey AS. Resistance training to reduce the malnutrition-inflammation complex syndrome of chronic kidney disease. Am J Kidney Dis. 2004 Apr;43(4):607–16.

## CV des principaux investigateurs

**Curriculum Vitae investigateur**

**Nom : CRISTOL**

Prénom : Jean-Paul

Date de naissance : 26/04/1959

Etablissement:  CHU Lapeyronie

Service / département: Laboratoire de Biochimie et Hormonologie

Adresse professionnelle: 371 avenue du doyen Gaston Giraud, 34295 Montpellier cedex 5

Téléphone:  04 67 33 83 15 e-Mail : :  jp-cristol@chu-montpellier.fr

Fonctions : Professeur des Universités - Praticien Hospitalier / Chef du Pôle Biologie Pathologie Titres : PU-PH

Affiliation éventuelle à un organisme de recherche :OUI, INSERM U1046-CNRS UMR9214

***Diplôme de docteur en Médecine***

Date d'obtention: 1989

Lieu d'obtention du diplôme : Montpellier

Numéro d’inscription à l’ordre: 34/07876 RPPS: 10003222667

***Diplômes de spécialité et Qualifications***

Intitulé précis: Certificat d'Etudes Spéciales en Néphrologie

Date d'obtention : 1990

Lieu d'obtention: Montpellier

Expérience en Recherche Clinique : Suivi de cohortes de patients IRC: PHRC national 2003 (UF7753), PHRC régional 2005 (UF7853), Appel d'Offres Cohortes 2011 (UF8851), Projet fil de l'eau 2013 (UF9125),

Etudes multicentriques sur le médicament: Etude SHARP (internationale), Etude FRENCH (nationale)

Etude d'évaluation de membranes de dialyse avec l'industrie: Hospal, Nipro, BBraun, Bellco, Fresenius Medical Care, Hemotech

***Principales publications***

1. Morena M, Jaussent I, Halkovich A, Dupuy AM, Bargnoux AS, Chenine L, Leray-Moragues H, Klouche K, Vernhet H, Canaud B, Cristol JP. Bone biomarkers help grading severity of coronary calcifications in non dialysis chronic kidney disease patients. PLoS One. 2012;7(5):e36175.
2. Morena M, Dupuy AM, Jaussent I, Vernhet H, Gahide G, Klouche K, Bargnoux AS, Delcourt C, Canaud B, Cristol JP. A cut-off value of plasma osteoprotegerin level may predict the presence of coronary artery calcifications in chronic kidney disease patients. Nephrol Dial Transplant. 2009 Nov;24(11):3389-97.
3. Badiou S, Cristol JP, Jaussent I, Terrier N, Morena M, Maurice F, Leray-Moragues H, Rivory JP, Chalabi L, Delcourt C, Canaud B, Dupuy AM. Fine-tuning of the prediction of mortality in hemodialysis patients by use of cytokine proteomic determination. Clin J Am Soc Nephrol., 2008 Mar;3(2):423-30.
4. Terrier N, Jaussent I, Dupuy AM, Morena M, Delcourt C, Chalabi L, Rouanet C,Canaud B, Cristol JP. Creatinine index and transthyretin as additive predictors of mortality in haemodialysis patients. Nephrol Dial Transplant., 2008 Jan;23(1):345-53.
5. Morena M, Terrier N, Jaussent I, Leray-Moragues H, Chalabi L, Rivory JP, Maurice F, Delcourt C, Cristol JP, Canaud B, Dupuy AM. "High osteoprotegerin level is predictive of mortality in hemodialysis patients: a potential link between bone and vascular biology". J Am Soc Nephrol., 2006 Jan;17(1):267-70.


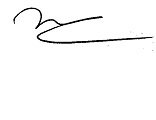
Date : 25/09/2015 Signature :

**Curriculum Vitae investigateur**

**Nom : MOURAD**

Prénom : Georges

Date de naissance : 22/03/1952

Etablissement:  CHU Lapeyronie

Service / département: Néphrologie

Adresse professionnelle: 371 avenue du doyen Gaston Giraud, 34295 Montpellier cedex 5

Téléphone:  04 67 33 84 76 e-Mail : :  g-mourad@chu-montpellier.fr

Fonctions : Professeur des Universités - Praticien Hospitalier / Chef de service

Titres :

***Diplôme de docteur en Médecine***

Date d'obtention: 20/02/1981

Lieu d'obtention du diplôme : Faculté de Médecine de Montpellier

Numéro RPPS: 10003226460

***Diplômes de spécialité et Qualifications***

Intitulé précis: Néphrologie

Date d'obtention : 01/03/1981

Lieu d'obtention: Faculté de Médecine de Montpellier

Expérience en Recherche Clinique : Oui

***Principales publications***

1. Du Cailar G, Oudot C, Fesler P, Mimran A, Bonnet B, Pernin V, Ribstein J, Mourad G. Left ventricular mass changes after renal transplantation: influence of dietary sodium and change in serum uric acid. Transplantation. 2014; 98(2):202-7
2. Mourad G, Minguet J, Pernin V, Garrigue V, Peraldi MN, Kessler M, Jacquelinet C, Couchoud C, Duny Y, Daurès JP. Similar patient survival following kidney allograft failure compared with non-transplanted patients. Kidney Int. 2014; 86(1):191-8.
3. Garrigue V, Szwarc I, Giral M, Soulillou JP, Legendre C, Kreis H, Kessler M, Ladrière M, Kamar N, Rostaing L, Morelon E, Buron F, Daguin P, Mourad G. Influence of anemia on patient and graft survival after renal transplantation: results from the French DIVAT cohort. Transplantation. 2014; 97(2):168-75.
4. Mourad G, Morelon E, Noël C, Glotz D, Lebranchu Y. The role of Thymoglobulin induction in kidney transplantation: an update. Clin Transplant. 2012; 26: 450-64.
5. Thierry A, Mourad G, Büchler M, et al. Steroid avoidance with early intensified dosing of enteric-coated mycophenolate sodium: a randomized multicentre trial in kidney transplant recipients. Nephrol Dial Transplant. 2012; 27 :3651-9
6. Kanaan N, Mourad G, Thervet E, Peeters P, Hourmant M, Vanrenterghem Y, De Meyer M, Mourad M, Maréchal C, Goffin E, Pirson Y. Recurrence and graft loss after kidney transplantation for henoch-schonlein purpura nephritis: a multicenter analysis. Clin J Am Soc Nephrol. 2011:1768-72.

Date : 02/10/2015 Signature :

**Curriculum Vitae investigateur**

**Nom : CHALABI**

Prénom : Lotfi

Date de naissance : 19/01/1960

Etablissement:  AIDER

Service / département: UDM

Adresse professionnelle: 191 avenue du doyen Gaston Giraud, 34295 Montpellier cedex 5

Téléphone:  04 30 78 13 05 e-Mail : :  lchalabi@aider.asso.fr

Fonctions : Néphrologue

Titres :

Affiliation éventuelle à un organisme de recherche :NON

***Diplôme de docteur en Médecine*** (Equivalence française du doctorat en Médecine)

Date d'obtention:  Octobre 1996

Lieu d'obtention du diplôme : Paris

Numéro d’inscription à l’ordre: 34/11308 RPPS: 10003242590

***Diplômes de spécialité et Qualifications***

Intitulé précis: Certificat d'Etudes Spéciales en Néphrologie

Date d'obtention : Octobre 1989

Lieu d'obtention: Montpellier

***Principales publications***

1. Bargnoux AS, Cristol JP, Jaussent I, Chalabi L, Bories P, Dion JJ, Henri P, Delage M, Dupuy AM, Badiou S, Canaud B, Morena M. Vitamin E-coated polysulfone membrane improved red blood cell antioxidant status in hemodialysis patients. J Nephrol. 2012 Sep 4:0.
2. Bargnoux AS, Morena M, Jaussent I, Maurice F, Chalabi L, Leray-Moragues H, Terrier N, Dupuy AM, Badiou S, Canaud B, Cristol JP. A combined index of cardiac biomarkers as a risk factor for early cardiovascular mortality in hemodialysis patients. Clin Chem Lab Med. 2013 Feb 12:1-10.
3. Patrier L, Dupuy AM, Granger Vallée A, Chalabi L, Morena M, Canaud B, Cristol JP. FGF-23 removal is improved by on-line high-efficiency hemodiafiltration compared to conventional high flux hemodialysis. J Nephrol. 2012 Apr 27:0.
4. Badiou S, Cristol JP, Jaussent I, Terrier N, Morena M, Maurice F, Leray-Moragues H, Rivory JP, Chalabi L, Delcourt C, Canaud B, Dupuy AM. Fine-tuning of the prediction of mortality in hemodialysis patients by use of cytokine proteomic determination. Clin J Am Soc Nephrol. 2008 Mar;3(2):423-30.
5. Terrier N, Jaussent I, Dupuy AM, Morena M, Delcourt C, Chalabi L, Rouanet C, Canaud B, Cristol JP. Creatinine index and transthyretin as additive predictors of mortality in haemodialysis patients. Nephrol Dial Transplant. 2008 Jan;23(1):345-53.


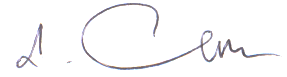


Date : 25/09/2015 Signature :

**Curriculum Vitae investigateur**

**Nom : LERAY-MORAGUES**

Prénom : Hélène

Date de naissance : 27/05/1965

Etablissement:  CHU Lapeyronie

Service / département: Néphrologie

Adresse professionnelle: 371 avenue du doyen Gaston Giraud, 34295 Montpellier cedex 5

Téléphone:  04 67 33 84 97 e-Mail : :  h-leray_moragues@chu-montpellier.fr

Fonctions : Praticien Hospitalier depuis le 01/07/1998

Titres :

***Diplôme de docteur en Médecine***

Date d'obtention: 04/11/1992

Lieu d'obtention du diplôme : Faculté de Médecine de Montpellier

Numéro RPPS: 10003225645 N° d'inscription au CNO : 34 - 8584

***Diplômes de spécialité et Qualifications***

Intitulé précis: Diplôme d’Etudes Spéciales de Néphrologie

Date d'obtention : 29/10/1992

Lieu d'obtention: Faculté de Médecine de Montpellier

DEA Biologie Santé: 1990-1991

Expérience en Recherche Clinique : Oui

***Principales publications***

1. Morena M, Jaussent I, Dupuy AM, Bargnoux AS, Kuster N, Chenine L, Leray-Moragues H, Klouche K, Vernhet H, Canaud B, Cristol JP. Osteoprotegerin and sclerostin in chronic kidney disease prior to dialysis: potential partners in vascular calcifications. Nephrol Dial Transplant. 2015 Apr 7. pii: gfv081.
2. Roubille F, Morena M, Leray-Moragues H, Canaud B, Cristol JP, Klouche K. Pharmacologic therapies for chronic and acute decompensated heart failure: specific insights on cardiorenal syndromes. Blood Purif. 2014;37 Suppl 2:20-33.
3. Canaud B, Granger Vallée A, Molinari N, Chenine L, Leray-Moragues H, Rodriguez A, Chalabi L, Morena M, Cristol JP. Creatinine index as a surrogate of lean body mass derived from urea Kt/V, pre-dialysis serum levels and anthropometric characteristics of haemodialysis patients. PLoS One. 2014 Mar 26;9(3):e93286.
4. Bargnoux AS, Morena M, Jaussent I, Maurice F, Chalabi L, Leray-Moragues H, Terrier N, Dupuy AM, Badiou S, Canaud B, Cristol JP. A combined index of cardiac biomarkers as a risk factor for early cardiovascular mortality in hemodialysis patients. Clin Chem Lab Med. 2013 Sep;51(9):1865-74.
5. Wilson P, Lertdumrongluk P, Leray-Moragués H, Chenine-Koualef L, Patrier L, Canaud B. Prevention and management of dialysis catheter complications in the intensive care unit. Blood Purif. 2012;34(2):194-9.
6. Morena M, Jaussent I, Halkovich A, Dupuy AM, Bargnoux AS, Chenine L, Leray-Moragues H, Klouche K, Vernhet H, Canaud B, Cristol JP. Bone biomarkers help grading severity of coronary calcifications in non dialysis chronic kidney disease patients. PLoS One. 2012;7(5):e36175.
7. Badiou S, Cristol JP, Jaussent I, Terrier N, Morena M, Maurice F, Leray-Moragues H, Rivory JP, Chalabi L, Delcourt C, Canaud B, Dupuy AM. Fine-tuning of the prediction of mortality in hemodialysis patients by use of cytokine proteomic determination. Clin J Am Soc Nephrol., 2008 Mar;3(2):423-30.


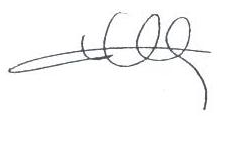
Date : 11/10/2015 Signature:

**Curriculum Vitae investigateur**

**Nom : CHENINE-KHOUALEF**

Prénom : Leila

Date de naissance : 26/01/1974

Etablissement:  CHU Lapeyronie

Service / département: Néphrologie

Adresse professionnelle: 371 avenue du doyen Gaston Giraud, 34295 Montpellier cedex 5

Téléphone: 04 67 33 84 97 e-Mail : : l-chenine@chu-montpellier.fr

Fonctions : Praticien Hospitalier depuis le 20/01/2013

***Diplôme de docteur en Médecine***

Date d'obtention: 04/11/1992

Lieu d'obtention du diplôme : Faculté de Médecine de Montpellier

Numéro RPPS: 10003225645 N° d'inscription au CNO : 34 - 8584

***Diplômes de spécialité et Qualifications***

Intitulé précis: AFS de néphrologie

Date d'obtention : 28/10/2006

Lieu d'obtention: Faculté de Médecine de Montpellier

***Principales publications***

1. Morena M, Jaussent I, Dupuy AM, Bargnoux AS, Kuster N, Chenine L, Leray-Moragues H, Klouche K, Vernhet H, Canaud B, Cristol JP. Osteoprotegerin and sclerostin in chronic kidney disease prior to dialysis: potential partners in vascular calcifications. Nephrol Dial Transplant. 2015 Apr 7. pii: gfv081.
2. • Morena M, Tuaillon E, Jaussent I, Rodriguez A, Chenine L, Vendrell JP, Cristol JP, Canaud B. Impairment in endothelial progenitor cells mobilization as a component of Malnutrition Inflammation Complex Syndrome: Which role for dialysis modalities? Congress of the European Renal Association - European Dialysis and Transplant Association. Paris, France, May 24-27, 2012.
3. Canaud B, Granger Vallée A, Molinari N, Chenine L, Leray-Moragues H, Rodriguez A, Chalabi L, Morena M, Cristol JP. Creatinine index as a surrogate of lean body mass derived from urea Kt/V, pre-dialysis serum levels and anthropometric characteristics of haemodialysis patients. PLoS One. 2014 Mar 26;9(3):e93286.
4. Wilson P, Lertdumrongluk P, Leray-Moragués H, Chenine-Koualef L, Patrier L, Canaud B. Prevention and management of dialysis catheter complications in the intensive care unit. Blood Purif. 2012;34(2):194-9.
5. Morena M, Jaussent I, Halkovich A, Dupuy AM, Bargnoux AS, Chenine L, Leray-Moragues H, Klouche K, Vernhet H, Canaud B, Cristol JP. Bone biomarkers help grading severity of coronary calcifications in non dialysis chronic kidney disease patients. PLoS One. 2012;7(5):e36175.

Date : 11/10/2015 Signature:
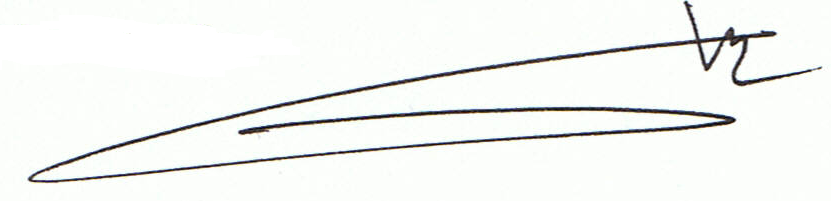


**Curriculum Vitae investigateur**

**NOM** : **HAYOT**

PRENOM : **Maurice**

DATE DE NAISSANCE : 02/ 03 /1963

Etablissement : CHU Arnaud de Villeuneuve

Service/département : Département de Physiologie Clinique, Hôpital Arnaud de Villeneuve – Adresse professionnelle : 371 Avenue du Doyen Giraud 34295 Montpellier cedex 5

Téléphone : 0467335908 Télécopie : 0467335923

E-Mail : m-hayot@chu-montpellier.fr

FONCTIONS : **Professeur des Universités - Praticien Hospitalier**

TITRE : **Docteur en Médecine MD – Docteur d’Université Ph D** (1993, Montpellier)

N° d'inscription au CNO : 34/08798

Numéro RPPS : 10003252243

SPECIALITES ET QUALIFICATIONS : **Pneumologue** (1993, Montpellier)

ORGANISME : **Université de Montpellier - CHRU Montpellier**

**Publications représentatives** *(5 références)* :

1. Maury J, Gouzi F, De Rigal P, Heraud N, Pincemail J, Molinari N, Pomiès P, Laoudj-Chenivesse D, Mercier J, Préfaut C, **Hayot M**. Heterogeneity of Systemic Oxidative Stress Profiles in COPD: A Potential Role of Gender**. Oxid Med Cell Longev**. 2015;2015:201843.

2. Pomiès P, Rodriguez J, Blaquière M, Sedraoui S, Gouzi F, Carnac G, Laoudj-Chenivesse D, Mercier J, Préfaut C, **Hayot M**. Reduced myotube diameter, atrophic signalling and elevated oxidative stress in cultured satellite cells from COPD patients. **J Cell Mol Med**. 2015 Jan;19(1):175-86.

3. Gouzi F, Préfaut C, Abdellaoui A, Roudier E, de Rigal P, Molinari N, Laoudj-Chenivesse D, Mercier J, Birot O, **Hayot M**. Blunted muscle angiogenic training-response in COPD patients versus sedentary controls. **Eur Respir J**. 2013 Apr;41(4):806-14.

4. Gouzi F, Maury J, Molinari N, Pomiès P, Mercier J, Préfaut C, **Hayot M**. Reference values for vastus lateralis fiber type proportion and fiber size. **J Appl Physiol** (1985). 2014 Jan 15;116(2):228

5. Gouzi F, Préfaut C, Abdellaoui A, Vuillemin A, Molinari N, Ninot G, Caris G, **Hayot M**. Evidence of an early physical activity reduction in chronic obstructive pulmonary disease patients. **Arch Phys Med Rehabil**. 2011 Oct;92(10):1611-1617.

Date : 22 octobre 2015

Signature :

**Curriculum Vitae investigateur**

**Nom : RAYNAL- RASCHILAS**

Prénom : Nathalie

Date de naissance : 28/03/1969

Etablissement:  AIDER

Service / département: Clinique des maladies rénales

Adresse professionnelle: 191 avenue du doyen Gaston Giraud, 34295 Montpellier cedex 5

Téléphone:  04 30 78 18 42 e-Mail : :  nraynal@aider.asso.fr

Fonctions : Médecin néphrologue salarié à temps plein depuis septembre 2004

***Diplôme de docteur en Médecine***

Date d'obtention: 01/03/1999

Lieu d'obtention du diplôme : Faculté de Médecine de Paris Saint Antoine

Numéro RPPS: 10001482958 N° d'inscription au CNO : 34 – 11214

***Diplômes de spécialité et Qualifications***

Intitulé précis: Diplôme d’Etudes Spéciales de Néphrologie


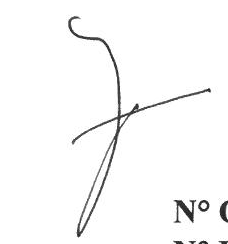


Date d'obtention : 01/03/1999

Lieu d'obtention: Faculté de Paris Saint Antoine

Date : 11/10/2015 Signature:

**Curriculum Vitae investigateur**

**Nom : PATRIER**

Prénom : Laure

Date de naissance : 09/07/1979

Etablissement:  AIDER

Service / département: Clinique des maladies rénales

Adresse professionnelle: Place du Professeur Debré, 30029 NIMES Cedex 9

Téléphone:  04 67 33 83 15 e-Mail : :l.patrier@aider.asso.fr

Fonctions : Fonctions : Médecin néphrologue salarié à temps plein depuis septembre Affiliation

***Diplôme de docteur en Médecine***

Date d'obtention: 14/12/2009

Lieu d'obtention du diplôme : Montpellier

RPPS: 10100098127

***Diplômes de spécialité et Qualifications***

Intitulé précis: DES de néphrologie

Date d'obtention : 25/10/2008

Lieu d'obtention: Montpellier

***Principales publications***

Date : 25/09/2015 Signature :

**Curriculum Vitae investigateur**

**Nom : MERCIER**

Prénom : Jacques

Date de naissance : 20/06/1956

Etablissement:  CHU Arnaud de villeuneuve / Université Montpellier

Service / département: Service de physiologie respiratoire

Adresse professionnelle: 371 avenue du doyen Gaston Giraud, 34295 Montpellier cedex 5

Téléphone:  04 67 33 59 08 e-Mail : :  jacques.mercier@univ-montp1.fr

Fonctions : Professeur des Universités-Coordonnateur du département de physiologie clinique au CHRU de Montpellier-Directeur U1046 INSERM/UMR 9214 « Physiologie et médecine expérimentale du coeur et des muscles-Vice-président chargé de la recherche à l’Université de Montpellier

Titres : PU-PH

Affiliation éventuelle à un organisme de recherche :OUI, INSERM U1046-CNRS UMR9214

**Titres** :

- 1984 Docteur en médecine

- 1990 Docteur de l'Université Montpellier 1

- 1991 Certificat d'Etudes Spécialisées en Pneumophtisiologie

- 1993 "Visiting Scholar" Université de Californie (Berkeley)

- 1995 Habiliation à diriger les recherches

**Diplômes de spécialité et Qualifications**

Intitulé précis: Pneumophtisiologie

Date d'obtention : 1991

Lieu d'obtention: Paris

Numéro ADELI ou RPPS : 10003216685

Numéro d’inscription à l’ordre: 34/6426

**Expérience en Recherche Clinique :**

1985: Certificat de méthodologie en médecine et santé publique

Investigateurs principal de plusieurs projets de recherche clinique

***Principales publications***

1: Lambert K, Coisy-Quivy M, Bisbal C, Sirvent P, Hugon G, Mercier J, Avignon A, Sultan A. Grape polyphenols supplementation reduces muscle atrophy in a mouse model of chronic inflammation. Nutrition. 2015 Oct;31(10):1275- 83.

2: Pomiès P, Rodriguez J, Blaquière M, Sedraoui S, Gouzi F, Carnac G, Laoudj-Chenivesse D, Mercier J, Préfaut C, Hayot M. Reduced myotube diameter, atrophic signalling and elevated oxidative stress in cultured satellite cells from COPD patients. J Cell Mol Med. 2015 Jan;19(1):175-86.

3: Passerieux E, Hayot M, Jaussent A, Carnac G, Gouzi F, Pillard F, Picot MC, Böcker K, Hugon G, Pincemail J, Defraigne JO, Verrips T, Mercier J, Laoudj-Chenivesse D. Effects of vitamin C, vitamin E, zinc gluconate, and selenomethionine supplementation on muscle function and oxidative stress biomarkers in patients with facioscapulohumeral dystrophy: a double-blind randomized controlled clinical trial. Free Radic Biol Med. 2015 Apr;81:158-69.

4: Fabre O, Breuker C, Amouzou C, Salehzada T, Kitzmann M, Mercier J, Bisbal C. Defects in TLR3 expression and RNase L activation lead to decreased MnSOD expression and insulin resistance in muscle cells of obese people. Cell Death Dis. 2014 Mar 20;5:e1136. doi: 10.1038/cddis.2014.104. PubMed PMID: 24651439; PubMed Central PMCID: PMC3973244.

5: Hokayem M, Blond E, Vidal H, Lambert K, Meugnier E, Feillet-Coudray C, Coudray C, Pesenti S, Luyton C, Lambert-Porcheron S, Sauvinet V, Fedou C, Brun JF, Rieusset J, Bisbal C, Sultan A, Mercier J, Goudable J, Dupuy AM, Cristol JP, Laville M, Avignon A. Grape polyphenols prevent fructose-induced oxidative stress and insulin resistance in first-degree relatives of type 2 diabetic patients. Diabetes Care. 2013 Jun;36(6):1454-61.

Date : 22/10/2015 Signature
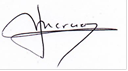

Supplement: S1 Protocol — (DOCX) [file pone.0200061.s002.docx]
