# Supplementary material for: Physical inactivity and protein energy wasting play independent roles in muscle weakness in maintenance haemodialysis patients
Source: PLoS One. 2018 Aug 1;13(8):e0200061. doi: 10.1371/journal.pone.0200061 (PMC6070183; doi:10.1371/journal.pone.0200061)
Supplement: S2 Protocol — (DOCX) [file pone.0200061.s003.docx]

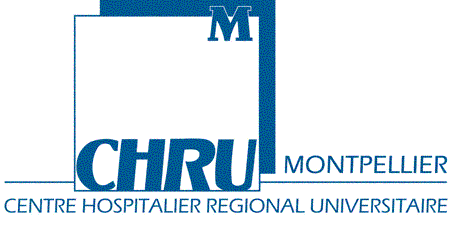


**trial study protocol**

Physical inactivity and protein energy wasting play independent roles in muscle weakness and atrophy in maintenance haemodialysis patients

CHRU de Montpellier

Centre Administratif André Bénech

191, avenue du Doyen Gaston Giraud

34295 Montpellier cedex 5

**N° ID RCB : 2015-A01854-45**

**Investigator**

CRISTOL, Jean-Paul

Email : jp-cristol@chu-montpellier.fr

Tél : 00 33 467 338 314

Table des matières

Table des matières

[1 General presentation 4](#_Toc502092189)

[1.1 Overview of the study 4](#_Toc502092190)

[1.2 Principal correspondents 4](#_Toc502092191)

[1.3 Summary of the study 5](#_Toc502092192)

[2 Background 7](#_Toc502092193)

[3 Objectives of the study 8](#_Toc502092194)

[3.1 Primary objective 8](#_Toc502092195)

[3.2 Secondary objectives 8](#_Toc502092196)

[4 Methodology 8](#_Toc502092197)

[4.1 Experimental design 8](#_Toc502092198)

[4.1.1 Type of study 8](#_Toc502092199)

[4.2 Subject selection 8](#_Toc502092200)

[4.2.1 Population 8](#_Toc502092201)

[4.2.2 Inclusion criteria 8](#_Toc502092202)

[4.2.3 Exclusion criteria 8](#_Toc502092203)

[4.3 Description of intervention (s) 9](#_Toc502092204)

[4.4 Judgment criteria 9](#_Toc502092205)

[4.4.1 Main judgment criteria 9](#_Toc502092206)

[4.4.2 Secondary endpoints 9](#_Toc502092207)

[4.5 Parameters collected and method of collection 9](#_Toc502092208)

[4.6 Statistics: 10](#_Toc502092209)

[4.6.1 Expected number of people to be included in the study 10](#_Toc502092210)

[4.6.2 Description of the planned statistical methods, including the schedule of planned interim analyzes 10](#_Toc502092211)

[5 Feasibility 11](#_Toc502092212)

[5.1 Recruitment potential 11](#_Toc502092213)

[5.2 Expertise / Availability of the teams 11](#_Toc502092214)

[5.3 Specific technical platform (s) and accessibility 11](#_Toc502092215)

[6 Practical procedure of the study 12](#_Toc502092216)

[6.1 Coordination of the study, location and role of each team 12](#_Toc502092217)

[6.2 Description of the care and follow-up of subjects 12](#_Toc502092218)

[6.2.1 Practical recruitment procedures 12](#_Toc502092219)

[6.2.2 Practical information and collection of non-opposition to participation in the study 12](#_Toc502092220)

[6.3 Patients visits 12](#_Toc502092221)

[6.4 Estimated timetable of the study 13](#_Toc502092222)

[7 Expected Results and Outlook 13](#_Toc502092223)

[8 Protection of persons 14](#_Toc502092224)

[8.1 Predictable benefits and risks known to those who are suitable for study 14](#_Toc502092225)

[8.1.1 Risks 14](#_Toc502092226)

[8.1.2 Risques 14](#_Toc502092227)

[8.2 Management of adverse events 14](#_Toc502092228)

[9 Data Management and Monitoring 18](#_Toc502092229)

[9.1 Monitoring 18](#_Toc502092230)

[9.2 inspection 18](#_Toc502092231)

[9.3 Collection and management of data 18](#_Toc502092232)

[9.4 Mention of the submission to the CNIL 18](#_Toc502092233)

[9.5 Storage and archiving 18](#_Toc502092234)

[10 Ethical and regulatory aspects 18](#_Toc502092235)

[11 Publication rules 19](#_Toc502092236)

[11.1 Study report 19](#_Toc502092237)

[11.2 Publication rules 19](#_Toc502092238)

[12 Bibliographies 19](#_Toc502092239)

# General presentation

## Overview of the study

**Title**

**Physical inactivity and protein energy wasting play independent roles in muscle weakness and atrophy in maintenance haemodialysis patients.**

N° ID-RCB : 2015-A01854-45

## Principal correspondents

**Promotor**

CHRU de MONTPELLIER

**Legal representative of the promoter**

Rodolphe BOURRET

Directeur Général - CHRU de MONTPELLIER

191, avenue du Doyen Gaston Giraud

[dg.secretariat@chu-montpellier.fr](mailto:dg.secretariat@chu-montpellier.fr)

**Coordonnateur (Equipe n°1)**

Jean-Paul CRISTOL

Statut : PU-PH

Spécialité : Biochimie

CHRU de Montpellier

Email : jp-cristol@chu-montpellier.fr

Tél : 04 67 33 83 14

## Summary of the study

**Full title of the project**

Physical inactivity and protein energy wasting play independent roles in muscle weakness and atrophy in maintenance haemodialysis patients.

**Background and rationale**

In chronic hemodialysis, uremic sarcopenia (with a prevalence of 20 to 50%), characterized by decreased mass and impaired muscle function is a significant mortality factor. The association of an increase in inflammation and oxidative stress, associated with kidney disease, defines the syndrome of Malnutrition, Inflammation and Atherogenesis (MIA syndrome). This syndrome is involved in the imbalance between anabolism and protein catabolism, which results in a decrease in the concentration of proteins necessary for muscle contraction. Thus muscle strength, dependent on muscle mass, could be limited by the MIA syndrome.

**Primary and secondary objectives**

- Main objective: To determine the contribution of the MIA syndrome in the reduction of muscle strength in chronic hemodialysis.

- Secondary objectives: In chronic hemodialysis determine:

The relationship between the maximal voluntary force (MVF) and muscle mass.

The relationship between MVF and lean mass.

The relationship between MVF and physical activity.

**Methods (study design, population, inclusion criteria, non inclusion, main and secondary evaluation criteria, number of topics to be included, statistical analysis ...)**

- Typology: Cross-sectional descriptive observational epidemiological study presenting minimal risks and constraints for the subject.

- Population (main eligibility criteria): chronic renal failure patients with chronic hemodialysis (> 3 months), aged between 18 and 90, with stable clinical status in the three months preceding inclusion.

- Criteria for judgment: The main criterion of judgment is the maximum voluntary force in Newton-meter.

**Procedure (number of visits, duration of inclusions, duration of follow-up, study schedule content of patient visits, brief description of the intervention):**

Two visits will be made.

The first visit (pre-inclusion) will verify the inclusion and non-inclusion criteria, inform the patient and obtain non-opposition to participate in the study.

The second visit will include:

- Measurement of MVF: Before the dialysis session

- Measurement of muscle mass by impedancemetry: after the dialysis session

- Collection of the biological parameters necessary for the estimation of the muscle mass and the MIA syndrome starting from the monthly report corresponding to the usual follow-up of the patient.

**Expected results and prospects**

Identifying the determinants of muscle strength in chronic hemodialysis will help to better understand the factors involved in the reduction of muscle strength. This will eventually allow to propose strategies based on the muscular retraining to prevent the appearance of a muscular deconditioning.

**Key words**

Key words: hemodialysis, muscle dysfunction, oxidative stress, inflammation.

# Background

4037/5000

Sarcopenia, defined as the association of loss of muscle mass and strength, is a geriatric process (1). The existence, in chronic hemodialysis, of a concomitant decrease in strength and muscle mass defines uremic sarcopenia, the prevalence of which is of the order of 20 to 50% (2). In this population, sarcopenia is a major prognostic marker of mortality (3).

The assessment of muscle mass is essential in hemodialysis because it is a reflection of the physical activity of patients and daily nutritional intake (4). Currently, two simple and quick bedside methods are used to evaluate the muscle mass of chronic hemodialysis patients (5,6). The first, the impedancemetry, measured by BCM (Body Composition Monitor, Fresenius Medical Care (6)), allows the measurement of lean mass (consisting of organs, skin, bones and muscles). The second, the creatinine index (CI) calculated from the parameters of hemodialysis and inter-dialytic production of creatinine, is a reflection of the metabolism of creatine and therefore of the muscle mass of hemodialysis patients (7). The limit of impedance measurement is the presence of an excess of inter-dialytic extracellular water. The measurement therefore needs to be standardized according to the periodicity of the dialysis sessions. The calculation of the creatinine index is modified by weekly changes in protein intake in patients (7). The isometric maximal voluntary force (MVF) is correlated with the surface of the muscle (r = 0.51) (8) in healthy subjects, and in some patients with chronic respiratory insufficiency (chronic obstructive pulmonary disease (9)). The measurement of muscle strength therefore appears as a new method of assessing muscle mass. Thus, in addition to allowing the functional assessment of the muscle (9), the isometric MVF of the quadriceps makes it possible to estimate the muscle mass of the patients outside certain neurological parameters (9). As a result, in chronic hemodialysis patients, MVF in combination with impedancemetry and the creatinine index would provide an accurate and rapid assessment of the patient's atrophy at the bedside.

The decrease in muscle mass in geriatrics results from an imbalance between anabolism and protein catabolism (1). Activation of protein catabolism is mediated by the ubiquitin-proteasome system (10). This pathway is activated by a reduction in the contractile activity of the muscle secondary to prolonged immobilization and by the reduction of physical activity of the patients (11). Anabolism is determined by physical activity and daily nutritional intake. In hemodialysis patients, in addition to decreased activity, undernutrition and immobilization, there are specific factors of muscle atrophy (11). Indeed, muscle atrophy may be the consequence of increased oxidative stress in this population (12) that results in activation of the ubiquitin-proteasome system (13). The syndrome of malnutrition, inflammation and atherogenesis (MIA syndrome), defined by an increase in inflammation and systemic oxidative stress (14), inducing an imbalance of the oxidation-reduction balance (15) could lead to a decrease the production of proteins necessary for muscle contraction (16). At the biological level, the markers of the MIA syndrome are the increase of the CRP and the decrease of the plasma albumin Thus the implication of the MIA in the imbalance between anabolism and protein catabolism, could explain its determining role in the appearance and the maintenance of muscle atrophy in chronic hemodialysis patients

WORKING HYPOTHESIS: In chronic hemodialysis, the MIA syndrome is a determining factor in the decrease in muscle mass estimated by the maximum voluntary force.

# Objectives of the study

## Primary objective

The primary objective of this study is to determine the contribution of MIA syndrome (increased CRP and decreased plasma albumin) in the decrease of MVF in chronic hemodialysis patients.

## Secondary objectives

In patients on chronic hemodialysis (HDC):

- The relationship between the MVF and the muscle mass appreciated by the creatinine index.

- The relationship between MVF and lean mass measured by impedance measurement.

- The correlation between MVF and physical activity estimated by the Voorrips score.

# Methodology

## Experimental design

### Type of study

Multidisciplinary cross-sectional descriptive observational epidemiological study presenting minimal risks or constraints for the subject.

## Subject selection

### Population

Patients with chronic renal failure at the end stage, chronic hemodialysis for at least 3 months.

### Inclusion criteria

| Age > à 18 years et < 90 years |
| --- |
| Chronic end-stage renal failure patient (GFR <15ml / min), with chronic dialysis hemodialysis treatment for at least 3 months. |
| Cardiovascular assessment less than two years old |
| Stable clinical status: In the three months prior to inclusion  -Absence of infection  -Absence of acute decompensation, chronic pathology (heart failure, respiratory failure, oedemato-ascitic decompensation).  -Absence of stroke. |
| Locomotive and neurological states of the lower limbs for the realization of the proposed functional tests. |
| Absence of congenital or genetic neurodegenerative muscular pathology |
| Collection of non-opposition to participation in the protocol |
| Obligation of affiliation or beneficiary of a social security scheme. |

### Exclusion criteria

| Major subject protected by law or unable to express his opposition to participation in the study according to Article L 1121-8 of the Public Health Code |
| --- |
| Vulnerable person according to article L.1121-6 of the Public Health Code |
| Subject not affiliated to a social security scheme, or not a beneficiary of such a schem |
| Chronic end-stage renal failure without adjunctive therapy |
| History of renal transplantation, in progress or having been resumed in hemodialysis in the 365 days preceding the day of inclusion. |
| Subject deprived of liberty by judicial or administrative decision |
| Cardiovascular contraindication to physical activity |
| Pregnant or breastfeeding women according to Article L.1121-5 of the Public Health Code |

## Description of intervention (s)

**Assessment of strength and muscle mass. :**

The measurement of the maximum voluntary force will be performed before the hemodialysis session

The measurement of muscle mass will be performed after the hemodialysis session

**Clinical data:**

From the clinical and biological parameters of the patient in his medical file: evaluation of his antecedents, comorbidities and systemic parameters of the MIA syndrome.

## Judgment criteria

### Main judgment criteria

In HDC patients, the multivariate analysis of the determinants of MVF (in Newton per meter): Muscle mass by impedancemetry and the Creatinine Index; the concentration of CRP and plasma albumin (MIA syndrome); physical activity by the Voorrips score.

### Secondary endpoints

In patients with HDC:

- the linear correlation (Pearson) between the creatinine index and the MVF.

- the linear correlation (Pearson) between the lean mass estimated by impedancemetry and the MVF.

- the linear correlation (Pearson) between the Voorrips score and the MVF.

- linear correlation (Pearson) between plasma albumin concentration and MVF

- linear correlation (Pearson) between hemoglobin concentration and MVF

## Parameters collected and method of collection

**Clinical parameters:**

Physical Activity Questionnaires:

The evaluation of the different types of activities (domestic, leisure, sports) will be made using the questionnaire of Baecke modified by Voorrips, which informs on the physical activity carried out in the previous year. This questionnaire has been used in several previous studies by INSERM Unit U1046.

Screening for comorbidities:

Charlson score, already validated in nephrology in chronic hemodialysis patients.

**Techniques for evaluating isometric maximal voluntary force and muscle mass:**

- Microfet2: Measurement of the maximum voluntary force by a portable dynamometer: Microfet-2; Hogan Scientific. The measurement will be in a sitting position on a hard plane, without back support, on an examination table. The angle between the thigh and the leg will be 90 °. The measurement will be made 2 cm above the external malleolus. The length of the lever arm is defined by the distance between the tibial plateaus and the area of ​​attachment of the strap to the leg.

**-** Bioanalytical impedancemetry: Multifrequency impedance measurement of body composition: Body Composition Monitor (BCM); Fresenius Medical Care. The patient will be lying down at rest for at least 15 minutes after the end of the hemodialysis session.

- Creatinine index: A measure of daily creatinine production by the creatinine index estimated from anthropometric data (age, sex), dialysis dose (Kt / V urea) and pre-dialytic creatinine level. The creatinine index derives from modeling the kinetics of creatinine (10). This model is recognized as reflecting the muscle mass and nutritional status of HDC patients (10).

- Techniques for assaying the systemic parameters of the MIA syndrome:

Determination of creatinine of albumin and CRP: respectively by enzymatic method, immunonephelometry and immunoturbidimetry.

## Statistics:

### Expected number of people to be included in the study

Considering a linear multivariate model with 5 covariates (albumin - CRP - Voorrips score - LBM - creatinine index), a risk of the first species of 5%, a desired power of 90% and a force of the effect of 0, As proposed by Cohen (Cohen Behavioral Sciences 1988) it will be necessary to include 110 patients. The calculation was carried out on the software R 3.1.1 (R Coreteam, Vienna, Austria) package pwr.

### Description of the planned statistical methods, including the schedule of planned interim analyzes

- Descriptive analysis and comparability of groups:

An initial descriptive analysis will be performed. For qualitative variables, this description will include the force. For quantitative variables, the description will include the size, mean, standard deviation, median, and extreme values by distribution.

- Analysis of the criteria of judgments:

The judgment criteria will be compared using a Student test. If the application conditions of the Student's test are not respected (normal distribution, equality of variances), a Mann-Whitney test will be used to compare these two groups.

Univariate correlations between the judgment criteria will be investigated using the Pearson or Spearman test, depending on whether or not the distribution of variables is normal. Multivariate analysis will be done by multiple linear regression.

A test will be considered significant when its degree of significance p is below the significance threshold of 5%.

Since no interim analysis is planned, a test will be considered significant when its degree of significance p is below the significance level of 5%. The analyzes will be carried out using the R version 2.13.0 software after the locking of the database and the approval of the Statistical Analysis Plan

# Feasibility

## Recruitment potential

The number of subjects likely to be included is 200 patients. Indeed, the number of hemodialysis patients in the four investigative centers is 200 patients. The expected rate of patients not meeting the inclusion and non-inclusion criteria is 30%. The foreseeable rate of refusal to participate in the protocol is 15%. In total, a possible inclusion number of 110 chronic hemodialysis subjects.

## Expertise / Availability of the teams

**The coordination of the test:** (steering committee, project leader) will be provided by Professor JP Cristol, principal investigator of the study and head of department of the Department of Biochemistry of the University Hospital Center, in collaboration with Prof. M. Hayot, Head of the Clinical Physiology Department of the University Hospital Center, and Prof. J. Mercier, Head of the INSERM U-1046 Research Team.

**The role of each team will be assigned before the study:**

-The recruitment will be carried out by all the investigating physicians of the CHRU / AIDER teams.

Team 1: Site CHU GCS Montpellier: Responsible Pr JP Cristol

Team 2: Site AIDER Valsiere: Head Dr N Raynal

Team 3: Site AIDER Montpellier: Head Dr. Chalabi

Team 4: Site AIDER Nîmes: Responsible Dr L Patrier

- The inclusion of patients and the collection, entry and processing of data will be provided by the investigating physicians of the CHRU / AIDER teams in each center.

- The final functional and clinical evaluations will be carried out by the CHRU / INSERM physiology department team.

- The quality control of the biological analyzes will be done by the biochemistry department.

- On the initiative of the coordinating physician, a meeting to set up the study will be scheduled at the beginning of the study as well as regular follow-up meetings.

## Specific technical platform (s) and accessibility

Team 1: Chronic Dialysis Center: GCS Lapeyronie's Medical Dialysis Unit (UDM)

Address: 371 avenue doyen Gaston Giraud, 34295 Montpellier cedex 5

Team 2: Chronic Dialysis Center: AIDER Autodialysis Unit (UAD) of La Valsiere

Address: 787 Rue de la Valsière, 34790 Grabels

Team 3: Chronic Dialysis Center: AIDER Lapeyronie Medicalized Dialysis Unit (UDM):

Address: 371 avenue doyen Gaston Giraud, 34295 Montpellier cedex 5

Team 4: Chronic Dialysis Center: AIDER Medicalized Dialysis Unit (UDM) in Nîmes:

Address: Professor Robert Debré Street, 30000 Nîmes

# Practical procedure of the study

## Coordination of the study, location and role of each team

**Places of realization of inclusions and muscular evaluations:**

The recruitment and inclusion of patients will be done in 4 centers of chronic hemodialysis of Languedoc Roussillon. The investigative centers are:

Chronic dialysis center: AIDER Autodialysis unit (UAD) of la Valsiere

Address: 787 Rue de la Valsière, 34790 Grabels

Chronic dialysis center: AIDER Lapeyronie's medical dialysis unit (UDM):

Address: 371 avenue doyen Gaston Giraud, 34295 Montpellier cedex 5

Chronic dialysis center: AIDER Medical dialysis unit (UDM) of Nîmes:

Address: Professor Robert Debré Street, 30000 Nîmes

Chronic dialysis center: GCS Lapeyronie's medical dialysis unit (UDM)

Address: 371 avenue doyen Gaston Giraud, 34295 Montpellier cedex 5

## Description of the care and follow-up of subjects

### Practical recruitment procedures

No examination will be done outside these 4 investigative centers. Each patient will be included in his usual hemodialysis center. For each patient, one of the investigator physicians will check if the patient meets all the criteria for inclusion and non-inclusion.

### Practical information and collection of non-opposition to participation in the study

The inclusion of chronic hemodialysis patients will be done during the dialysis session. Patients will be informed by one of the investigators in advance by an information note and an oral explanation. This information visit will correspond to the pre-inclusion visit V1.After a reflection period of one week, if the patient agrees to participate, the investigating physician will collect his written and signed informed consent and the patient will be included. This second visit will correspond to the visit V2

## Patients visits

Visit V1: Verification of inclusion and non-inclusion criteria, patient information, clinical examination.

Visit V2:

Collection of informed consent written and signed by the subject participating in the protocol and by one of the investigators of the study. (An original will be kept by the investigator, an original will be given to the research participant.)

Before the hemodialysis session: measurement of the maximum voluntary force.

After the hemodialysis session: measurement of lean mass.

Note :

- The measurement of the maximum voluntary force of each patient is performed before the dialysis session to avoid any additional fatigue secondary to dialysis. It will not delay the care of the patient, since it will be performed during the preparation of the dialysis station by the nursing staff (assembly and disinfection of the machine). The evaluation of muscle strength is currently recommended in chronic hemodialysis because it is necessary to screen for uremic sarcopenia.

- Measurement of lean mass by impedancemetry will be done 15 minutes after the end of the patient's dialysis session, in order to allow a return to equilibrium between the various fluid sectors (interstitial - vascular - intracellular) (6). In order not to delay the return home of the patient the measurement will be carried out immediately after the cessation of extracorporeal circulation (ECC) and closure of the vascular approach (1 / If arteriovenous fistula: manual compression 2 / If dialysis catheters : closure and occlusive dressing). The use of an impedancemetre in chronic hemodialysis is recommended because it is a necessary tool on the one hand for the estimation of dry weight in chronic hemodialysis and on the other hand for screening for uremic sarcopenia.

- The biological parameters necessary for calculating the creatinine index and the MIA syndrome estimate will be collected from the monthly balance sheet of the month in which the measurements of the maximum voluntary force and lean mass of each patient were taken.

NB

The biological blood parameters analyzed in this study are part of the monthly biological assessment (or half-yearly) corresponding to the usual follow-up of the hemodialysis patient and do not therefore require additional blood sampling.

## Estimated timetable of the study

Obtaining regulatory notices: 3 months (M0 - M3)

Inclusion period: 6 months

Duration of Follow-up: Not applicable

End of monitoring: Not applicable

Duration of the freezing of the database and statistical analysis / valuation: 2 months

Drafting of the final report: 2 months

Communication date of the final report: December 2016

Publication: January 2017

Estimated duration of the study: 13 months (from M0 to M13)

# Expected Results and Outlook

**ORIGINALITY AND INNOVATIVE CHARACTER:**

The originality of this project is to jointly study the mass and muscle strength of patients on chronic hemodialysis (HDC). First, the assessment of muscle strength is based on a simple, fast, and non-invasive screening tool that will establish MVF values based on the age, weight, height, and sex of hemodialysis patients. chronic. Secondly, the evaluation of the muscular mass, delicate in hemodialysis because of the excess of extracellular water will be done by two complementary methods, by impedancemetry and by the creatinine index.

In the chronic hemodialysis patient, muscular dysfunction is a significant factor in morbidity and mortality and impairment of quality of life (17). Few studies have investigated the characterization of muscle dysfunction and the determinants behind these clinical abnormalities. In these patients, both lean body mass (LBM) loss of muscle mass (18) and impaired muscle function (19) are observed, leading to decreased exercise capacity (4). ). Inflammatory syndrome and oxidative stress in malnutrition-inflammation-atherogenesis (MIA) syndrome (20) are two important players in this muscle dysfunction (19) (21).

Prospective cohort of chronic hemodialysis patients: Evaluate, by prospective follow-up, the survival of chronic hemodialysis patients according to their strength and muscle mass.

Effort Retraining in Chronic Hemodialysis Patients: Evaluate the muscle strength and muscle mass of chronic hemodialysis patients after muscle reconditioning**.**

# Protection of persons

## Predictable benefits and risks known to those who are suitable for study

### Risks

**EXPECTED BENEFITS FOR PATIENT AND / OR PUBLIC HEALTH:**

Identifying precisely the determinants of muscle strength in HDC will help to better understand the factors involved in decreasing muscle strength in chronic hemodialysis patients.

Finally, a better understanding of the mechanisms of muscular dysfunction will allow us to propose early preventive strategies based on muscular retraining to prevent the onset of muscular deconditioning.

**BENEFICES ATTENDUS POUR LE PATIENT ET/OU POUR LA SANTE PUBLIQUE :**

### Risques

The risks of biomedical research:

1 / Use of Microfet2: Risk of muscle cramps: The assessment of maximum strength by Microfet2 can be accompanied by muscle cramps. However, this risk is limited because of the speed of measurement. Moreover, this measurement is performed before the dialysis session, which avoids an increase in cramps secondary to dialysis.

2 / Use of an impedance meter: The measurement of the body composition by impedancemetry is carried out 15 min after the disconnection of the patient, which supposes a return to the delayed home and a burden of the increased renal disease. However, after disconnection, the patient is required to lie down for an incompressible period (on average 15-20 minutes), the time that the health care staff ensures the good closure of the vascular approach and measures certain constants (frequency heart rate, blood pressure). The measure of body composition is planned during this incompressible period, the return to the home will not be delayed.

## Management of adverse events

The research will be carried out in accordance with the French regulations in force, in particular the provisions relating to biomedical research: European Directive 2001/20 / CE, Public Health Law of 9 August 2004, Decree of application n ° 2006-477 of the April 26, 2006.

Regarding the vigilance of the project, the responsibilities of the investigator and the sponsor, the declaration of serious adverse events, the annual safety reports will be monitored and carried out in accordance with the regulations.

Definitions

Adverse Event (AE): Any harmful event occurring in a person who is amenable to biomedical research that this event is related or not to the research or the product to which this research relates.

This definition applies to:

Any exacerbation of a pre-existing disease,

Any increase in the frequency or severity of a pre-existing intermittent event / clinical condition, any symptom / disease discovered after the start of the study, even if it existed before inclusion of the subject in the trial.

Any symptom / disease present at the inclusion of the subject and worsening during the test.

Adverse effect (EfI): any adverse and unwanted reaction attributable to one or more characteristic parameters of the research protocol (procedures, methods, procedures performed or products being researched or used for research purposes).

For each adverse event, the investigator and the sponsor evaluate its causal link with the research. Any adverse event considered by the investigator to have a scientifically reasonable causal link to the research is classified as an adverse event. The term "scientifically reasonable causation" generally means that there is evidence or argument to suggest, from a scientific point of view, a causal relationship between the observed adverse and undesirable reaction and the research.

Unexpected side-effects (EfI-I): any adverse effect whose nature, severity or evolution does not match the information contained in the reference document (Summary of Product Characteristics, Investigator's Brochure ... or instructions for use of DM)

New Developments: Any new safety data, which may lead to a re-evaluation of the benefit / risk ratio of the investigational or experimental drug, or which may be sufficient to consider modifications in the administration of the investigational drug, in the conduct of the research.

Event or serious adverse reaction (SAE): A serious adverse event or adverse event is an event (or effect):

- whose evolution is fatal,

- endangers the life of the person who is suitable for research,

- which causes a disability or significant or long-term disability,

- which causes hospitalization or prolongation of hospitalization

- which results in an abnormality or congenital malformation

- any other event not satisfying the qualifications listed above, but which may be considered as "potentially serious", particularly certain biological abnormalities

- medically relevant event as determined by the investigator

- an event requiring medical intervention to prevent the progression towards one of the aforementioned states.

For example, these events may be intensive treatment in hospital emergencies or at the home of the research participant for allergic bronchospasm, seizure, or bleeding disorders.

The term "life-threatening" is reserved for an immediate life-threatening threat at the time of the adverse event, regardless of the consequences of corrective or palliative therapy.

Certain circumstances requiring hospitalization do not fall under the criterion of severity "hospitalization / prolongation of hospitalization" as:

- admission for social or administrative reasons

- hospitalization predefined by the protocol

- hospitalization for medical or surgical treatment programmed before the search

- passage to day hospital

Serious events not to report immediately

Identify serious adverse events that do not require immediate reporting in agreement with the health authorities.

Listing of the expected side effects with this protocol

Responsibilities of the investigator

Methods of collecting adverse events

All adverse events will be noted on the adverse event collection forms in the observation booklet. Each adverse event observed will be recorded individually. All adverse events should be graded in intensity.

Statement of the EIG

The investigator evaluates each adverse event in terms of its severity.

The investigator shall promptly notify the sponsor, from the day of its knowledge, of all serious adverse events occurring in the trial, except those identified in the protocol as not requiring notification. immediate.

This initial notification is made by fax to the following address:

Directorate of Research and Innovation

Pharmacovigilance of Clinical Trials

04 67 33 91 72

This initial notification is the subject of a written report and must be followed if necessary by one or more detailed written report (s).

Evaluation of causality:

The investigator must evaluate the causal link of the events with the research (experimental drug, comparator, surgical technique ...). The causal link is binary (connected / not connected).

Proponent's responsibilities

EIGI Statements and New Developments

The sponsor must evaluate the causal link between the serious adverse event and the research. It assesses whether the adverse event is expected or unexpected with the help of the reference document.

He declares within the statutory deadlines all the new and serious and unexpected undesirable effects to the EMA (seizure EudraVigilance, European pharmacovigilance database), to the competent health authorities and to the ethics committees concerned and informs the investigators.

The regulatory declaration is made within a maximum period of:

- 7 calendar days for unexpected serious adverse or life-threatening adverse reactions. In this case, additional relevant information must be sought and transmitted within a further 8 days.

- 15 calendar days for all other unexpected serious effects. Likewise additional relevant information must be sought and transmitted within a further period of 8 days.

Annual safety report

On the anniversary date of the test authorization issued by the Health Authorities as part of the testing of a health product, or on the anniversary date of the first inclusion in other biomedical research, the sponsor writes an annual report security comprising:

- the list of serious adverse reactions that may be related to the research, including unexpected and expected serious effects.

- a concise and critical analysis of the safety of patients amenable to research.

This report may be submitted to the coordinating investigator for approval.

This report is sent to the relevant authorities and ethics committees within 60 days of the anniversary date.

Independent monitoring committee (if not set up, put the justifications below)

An independent trial monitoring committee will be formed as part of this study. It is an advisory committee to ensure the protection of patients, to ensure that the trial is conducted ethically, to assess the benefit / risk ratio of the trial and to ensure independent review. scientific results in progress or at the end of the test.

Its members, competent in the field of clinical trials (pathology, methodology ...), are not involved in the study. The selection of the members of the Independent Monitoring Committee is made by the sponsor in collaboration with the coordinating investigator.

They are appointed and mandated by the sponsor for the duration of the study. They commit to their participation on a voluntary basis as well as to respect the confidentiality of the data.

The Independent Monitoring Committee receives the successive versions of the protocol, the Annual Safety Reports. It may be requested at any time by the sponsor if an unexpected serious adverse reaction or a serious adverse event presents a particular difficulty of analysis, if data likely to modify the benefit / risk ratio appear during the study.

The Independent Monitoring Committee analyzes the data transmitted to it, may request additional information. It issues opinions and recommendations on the future of the study (prosecution, amendments, judgment ...).

It will be composed of people outside the research of which necessarily:

- a clinician specialized in the pathology studied,

- a pharmacologist / pharmaco vigilant

- a methodologist / biostatistician.

The composition and operating rules (pace of meetings ...) are defined in the charter of the Independent Monitoring Committee.

# Data Management and Monitoring

## Monitoring

The project will be monitored by a clinical research assistant delegated by the promoter (ARC promoter). An adapted monitoring will be implemented according to a grid of risks related to the project. According to this grid, the ARC promoter will carry out regular visits to the project's investigation centers (implementation visit, follow-up according to the rhythm of the inclusions and a closing visit). Any visit will be the subject of a monitoring report by written report (traceability of visits).

## inspection

Investigators agree to comply with the requirements of the sponsor and the Competent Authority with respect to an audit or inspection of the research.The audit can be applied at all stages of the research, from the development of the protocol to the publication of the results and the classification of the data used or produced as part of the research.

## Collection and management of data

The participant will only be identified by a unique identification number, the first letter of the name, the first letter of the first name and the year of birth. An identification list of subjects will be kept in the investigator's file. The investigator will ensure that the anonymity of each person participating in the study is guaranteed. Information will be collected for each participant on a standardized observation book completed by the investigator or co-investigator.Source documents: The source documents are the original documents, the data and the files, from which the data concerning the research participants are reported in the observation form. The investigator undertakes to allow direct access to the source data of the study during inspection, audit or inspection visits.

## Mention of the submission to the CNIL

The information collected during this study may be subject to computer processing. The file will be produced in accordance with the procedure MR001 of the CNIL (National Commission of Computing and Freedoms) applicable. National Commission of Computing and Freedoms).

## Storage and archiving

The closing of the trial including the closure of the centers will be done in accordance with Good Clinical Practice and ICH. Medical records, administrative records, and notebooks will be kept for the duration of the study in the department and archived for a minimum of 15 years.

# Ethical and regulatory aspects

The research will be carried out in accordance with the French regulations in force, in particular the provisions relating to biomedical research of the Public Health Code, articles L1121-1 and following (law of public health of August 9, 2004), the laws of Bioethics the data Protection Act, the declaration of Helsinki and Good Clinical Practice.

Committee for the Protection of Persons (CPP):

Before the implementation of the research, the developer will submit the draft to the opinion of the people of South Mediterranean I. Protection Committee (Under RBM)

Competent authority (ANSM):

Before making or to perform biomedical research, the sponsor of this research address a request for authorization to the competent authority. (As part of RBM)

Information and consent of participants:

Prior to carrying out a biomedical research on a person, the free, informed and written consent of the subject must be collected after he has been informed by the investigator during a prior visit (V1) and a delay of sufficient reflection of a week.

The information intended for the participants of the trial must include all the elements defined in the public health law of 9 August 2004 and must be written in a simple manner, in a language understandable by (the parents and) the participant. After having read it, the consent form must be dated and signed personally by the research participant and the investigator (an original will be kept by the investigator, an original will be given to the research participant).

Insurance: The University Hospital of Montpellier, promoter of the study, subscribes for the whole duration of the study an insurance guaranteeing its own civil liability as well as that of any intervener involved in the realization of the test, independently of the nature of the links between stakeholders and the proponent. (As part of RBM).

# Publication rules

## Study report

A final report dated and signed by the investigator will be forwarded to the sponsor, who will forward it to the competent authorities within 12 months of the end of the study.

## Publication rules

Any written or oral communication of the research results must receive the prior agreement of the coordinating investigator and the sponsor.

The University Hospital of Montpellier is the owner of the data and no use or transmission to a third party can be carried out without its prior agreement.

The University Hospital of Montpellier, promoter of the research, must be mentioned in the publications according to the following address writing model:

CHU Montpellier, department / service, city, F-postal code, country.

# Bibliographies

1. Cruz-Jentoft AJ, Baeyens JP, Bauer JM, Boirie Y, Cederholm T, Landi F, et al. Sarcopenia: European consensus on definition and diagnosis: Report of the European Working Group on Sarcopenia in Older People. Age Ageing. 2010 Jul;39(4):412–23.

2. Isoyama N, Qureshi AR, Avesani CM, Lindholm B, Bàràny P, Heimbürger O, et al. Comparative associations of muscle mass and muscle strength with mortality in dialysis patients. Clin J Am Soc Nephrol. 2014 Oct 7;9(10):1720–8.

3. Pereira RA, Cordeiro AC, Avesani CM, Carrero JJ, Lindholm B, Amparo FC, et al. Sarcopenia in chronic kidney disease on conservative therapy: prevalence and association with mortality. Nephrol Dial Transplant. 2015 Oct;30(10):1718–25.

4. Domański M, Ciechanowski K. Sarcopenia: a major challenge in elderly patients with end-stage renal disease. J Aging Res. 2012;2012:754739.

5. Terrier N, Jaussent I, Dupuy A-M, Morena M, Delcourt C, Chalabi L, et al. Creatinine index and transthyretin as additive predictors of mortality in haemodialysis patients. Nephrol Dial Transplant. 2008 Jan;23(1):345–53.

6. Marcelli D, Usvyat LA, Kotanko P, Bayh I, Canaud B, Etter M, et al. Body Composition and Survival in Dialysis Patients: Results from an International Cohort Study. Clin J Am Soc Nephrol. 2015 Apr 21;

7. Canaud B, Granger Vallée A, Molinari N, Chenine L, Leray-Moragues H, Rodriguez A, et al. Creatinine index as a surrogate of lean body mass derived from urea Kt/V, pre-dialysis serum levels and anthropometric characteristics of haemodialysis patients. PLoS ONE. 2014;9(3):e93286.

8. Maughan RJ, Watson JS, Weir J. Strength and cross-sectional area of human skeletal muscle. J Physiol (Lond). 1983 May;338:37–49.

9. Seymour JM, Spruit MA, Hopkinson NS, Natanek SA, Man WD-C, Jackson A, et al. The prevalence of quadriceps weakness in COPD and the relationship with disease severity. Eur Respir J. 2010 Jul;36(1):81–8.

10. Workeneh BT, Mitch WE. Review of muscle wasting associated with chronic kidney disease. Am J Clin Nutr. 2010 Apr;91(4):1128S – 1132S.

11. Panaye M, Kolko-Labadens A, Lasseur C, Paillasseur J-L, Guillodo MP, Levannier M, et al. Phenotypes influencing low physical activity in maintenance dialysis. J Ren Nutr. 2015 Jan;25(1):31–9.

12. Baptista G, Dupuy A-M, Jaussent A, Durant R, Ventura E, Sauguet P, et al. Low-grade chronic inflammation and superoxide anion production by NADPH oxidase are the main determinants of physical frailty in older adults. Free Radic Res. 2012 Sep;46(9):1108–14.

13. Rajan VR, Mitch WE. Muscle wasting in chronic kidney disease: the role of the ubiquitin proteasome system and its clinical impact. Pediatr Nephrol. 2008 Apr;23(4):527–35.

14. Stenvinkel P, Heimbürger O, Lindholm B, Kaysen GA, Bergström J. Are there two types of malnutrition in chronic renal failure? Evidence for relationships between malnutrition, inflammation and atherosclerosis (MIA syndrome). Nephrol Dial Transplant. 2000 Jul;15(7):953–60.

15. Yazdi PG, Moradi H, Yang J-Y, Wang PH, Vaziri ND. Skeletal muscle mitochondrial depletion and dysfunction in chronic kidney disease. Int J Clin Exp Med. 2013;6(7):532–9.

16. Adey D, Kumar R, McCarthy JT, Nair KS. Reduced synthesis of muscle proteins in chronic renal failure. Am J Physiol Endocrinol Metab. 2000 Feb;278(2):E219–25.

17. Noori N, Kopple JD, Kovesdy CP, Feroze U, Sim JJ, Murali SB, et al. Mid-arm muscle circumference and quality of life and survival in maintenance hemodialysis patients. Clin J Am Soc Nephrol. 2010 Dec;5(12):2258–68.

18. Terrier N, Senécal L, Dupuy A-M, Jaussent I, Delcourt C, Leray H, et al. Association between novel indices of malnutrition-inflammation complex syndrome and cardiovascular disease in hemodialysis patients. Hemodial Int. 2005 Apr;9(2):159–68.

19. Fahal IH, Bell GM, Bone JM, Edwards RH. Physiological abnormalities of skeletal muscle in dialysis patients. Nephrol Dial Transplant. 1997 Jan;12(1):119–27.

20. Desmeules S, Lévesque R, Jaussent I, Leray-Moragues H, Chalabi L, Canaud B. Creatinine index and lean body mass are excellent predictors of long-term survival in haemodiafiltration patients. Nephrol Dial Transplant. 2004 May;19(5):1182–9.

21. Castaneda C, Gordon PL, Parker RC, Uhlin KL, Roubenoff R, Levey AS. Resistance training to reduce the malnutrition-inflammation complex syndrome of chronic kidney disease. Am J Kidney Dis. 2004 Apr;43(4):607–16.
